# Identifying the origin of springs in weathered-fractured crystalline aquifers using a hydrogeophysical approach

**DOI:** 10.1038/s41598-024-63748-8

**Published:** 2024-06-05

**Authors:** Kouassi Jean-Michel Kouassi, Patrick Lachassagne, Oi Mangoua Jules Mangoua, Abé Parfait Sombo, Brou Dibi

**Affiliations:** 1 Laboratory of Environmental Sciences and Technologies, Univ. Jean Lorougnon Guédé, Daloa, Ivory Coast; 2 HSM, Univ. Montpellier, CNRS, IRD, IMT Mines Alès, Montpellier, France

**Keywords:** Hydrology, Environmental sciences, Hydrogeology, Geophysics

## Abstract

Over the last few decades, important advances have been made in the development of relevant hydrogeological conceptual models for crystalline aquifers, and notably for weathered-fractured crystalline aquifers. Paradoxically and contrary to other types of aquifers, these researches never aimed at characterizing springs, the places were groundwater naturally outflows from such aquifers. With such an objective, our methodological approach consisted first of a lithological and hydrogeological description of the aquifer system based on borehole data and outcrops in a representative weathered-fractured crystalline aquifer (Daloa, Ivory Coast). Next, electrical resistivity tomography (ERT) has been used (after validating the appropriate inversion method) to provide the imagery of the weathering profile both below the plateaus and in the valleys where the springs outflow. Piezometric and river discharge data were also processed notably to determine the direction of groundwater flow. Results demonstrate unambiguously that the isalterites aquifer supplies the springs, and that the underlying fractured layer is not directly implied in this supply. ERT combined with borehole and field lithological data also shows that the lateritic formations (alloterites) present near surface below the plateaus, as well as the upper part of the isalterites, were eroded in the valleys, but not deep enough to let the fractured layer outcrop. This conceptual model for springs not only provides a basis for characterizing such complex aquifers, but also provides technical guidance for spring catchment and groundwater protection in these crystalline areas.

## Introduction

Water is essential for life, both within ecosystems and for humanity. Yet millions of people around the world struggle daily to find drinking water to meet their basic needs^1,2^. Groundwater is increasingly used worldwide due to the impact of climate change and anthropogenic activities on surface water resources, both in terms of quantity and quality. Groundwater covers around 43% of irrigation water needs^3^, and also makes an important contribute to hydration, sanitation and hygiene in many parts of the world^2^. Although groundwater is increasingly in demand throughout the world, its exploitation and management are often difficult and costly due to the complexity of the aquifer systems that contain it, notably those that are fractured^4–8^. The African continent is no exception. Moreover, over a large part of its surface, the only available resource of groundwater comes from aquifers from granitic and metamorphic rocks known as "crystalline"^4,5^. Worldwide, these crystalline aquifers make up more than 20% of the world's land surface^5,9,10^. They have a fracture permeability^5^ and, as a result, their structure and functioning are less well understood than those of other types of aquifers, notably porous aquifers, making it difficult and costly to exploit the groundwater they contain^5^.

In West Africa, groundwater accounts for a significant and steadily increasing proportion of the rural population's water supply, particularly in the crystalline context. Indeed, the groundwater contained in these crystalline aquifers is geographically well distributed^11–15^ and offers an alternative to surface water resources, which are subject to contamination and are unavailable all or part of the year, particularly in semi-arid regions. These groundwaters resources are generally accessed by drilling. However, the complexity of crystalline aquifers means that between 25 and 60% of boreholes drilled are "dry"^16–18^. However, these West African crystalline rock regions also contain numerous springs^19,20^, which could also be exploited to supply the population with water. Springs are natural groundwater outflows that appear locally or diffusely on the surface of the ground. They play an important role in socio-economic development in many parts of the world, where they are tapped for a variety of uses. Paradoxically, in crystalline aquifers, the processes and hydrogeological context that explains why groundwater outflows at springs is less well known than that of boreholes^5^. It is therefore important to improve the hydrogeological conceptual model of crystalline aquifers in areas where springs are present, in order to characterize the type of springs, their context of emergence, their vulnerability to contamination and to verify their seasonal sustainability^21,22^.

Over the last two decades^5,23^, the hydrogeological conceptual model of crystalline aquifers has evolved considerably, from concepts invoking fractures of tectonic origin to a concept of fractures linked to weathering processes. Methods for characterizing the structure and functioning of these hydrosystems have evolved accordingly^4,5,16,21,23–26^. However, these methods do not provide relevant information on the origin and properties of springs in this type of geological environment. In this research, we therefore explore the use of electrical geophysical methods to develop the conceptual model of springs in a crystalline rock context, using an integrated approach in which geological, hydrogeological and geophysical data are jointly interpreted.

Over the last few decades, numerous advances have been made in the development of representative conceptual models of crystalline aquifers based on geophysical methods^27–34^. However, these researches never addressed the issue of spring characterization. Electrical resistivity tomography (ERT) methods are widely used in this type of context, as they provide 2D information, or even quasi 3D in the case of serial profiles, and as there is a good correlation between geophysical facies and those of the weathering profile^35–38^. The complexity of crystalline environments gives rise to biases that severely limit the validity of 1D methods^28,37,39,40^ which, for technical and low-cost reasons, are unfortunately still often used.

Building on feedback from previous research, this study aims to assess the effectiveness of ERT to characterize the context of groundwater springs in crystalline rock environments. Specifically, we aim to: (i) assess the impact of geophysical data inversion methods on the quality of hydrogeological models in a crystalline rock context, and (ii) leverage a large geological, hydrogeological and geophysical data set to improve the conceptualization of crystalline aquifers. To meet these objectives, we first described the lithology and hydrogeology of the aquifer system using borehole and outcrop data. The results of geophysical inversions were then calibrated on these geological data to provide a 2D conceptualization of the weathering profile. Piezometric data were also processed to determine the groundwater flow direction in the aquifer.

**Figure 1:**
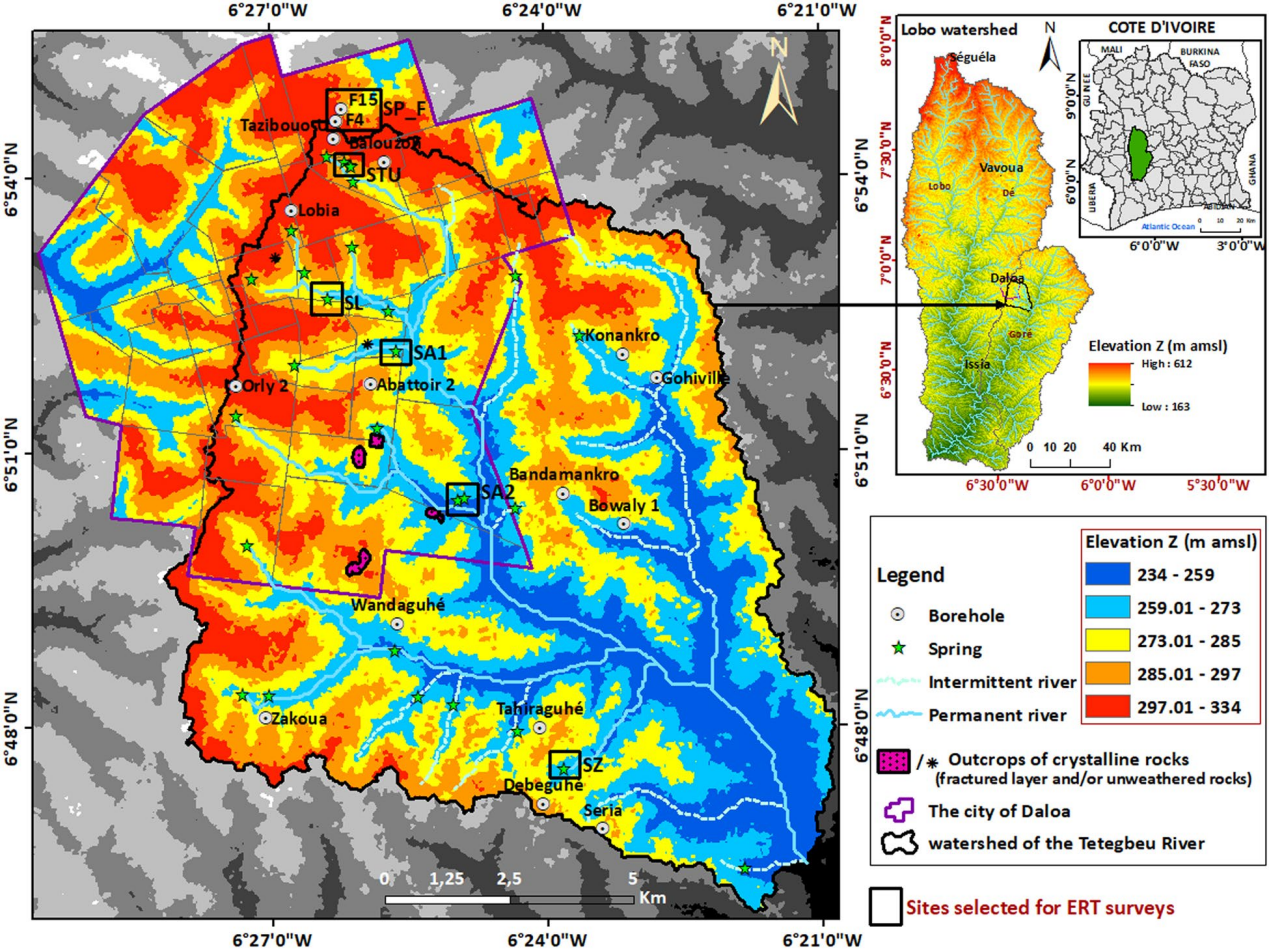
Location and topographic map of the study area. Location of the sites selected for ERT surveys. SP_F: site of ERT profiles SP_F4 and SP_F15 (the lithology of boreholes F4 and F15 was used to calibrate the SP_F4 and SP_F15 ERT profiles), STU: site of ERT profiles STU1 and STU2, SL: site of ERT profile SL, SA1: site of ERT profile SA1, SA2: site of ERT profiles SA2 and SZ: site of ERT profile SZ. The electrical cross sections for the SL, SA1, SA2 and SZ profiles are shown in Appendix 4 and 5 in the Complementary Materials. This graph was produced using ArcGIS 10.2.

The research is being carried out on the aquifers from the catchment area of the Tétégbeu River (Fig. 1), a tributary of the Lobo River (Center-West, Ivory Coast). The Tétégbeu River starts flowing in the municipality of Daloa and drains an area of around 140 km^2^. The climate belongs to the attenuated transitional equatorial regime, with average annual rainfall and temperature of 1200 mm and 26 °C respectively (see Appendix 2 in the Complementary Materials). The topography of the basin is relatively homogeneous (Fig. 1), with predominantly flat, gently sloping plateaus incised by the hydrographic network. The subsoil in the research area is composed of Middle Precambrian formations, dominated by granites and granodiorites^41^, with granodiorites accounting for 85% of the surface area (see Appendix 1 in the Complementary Materials). These plutonic rocks were deeply weathered during the Mesozoic to Quaternary eras, and are therefore covered by weathering profiles several tens of meters thick.

## Results

### Description of the weathering profile at borehole scale

**Figure 2:**
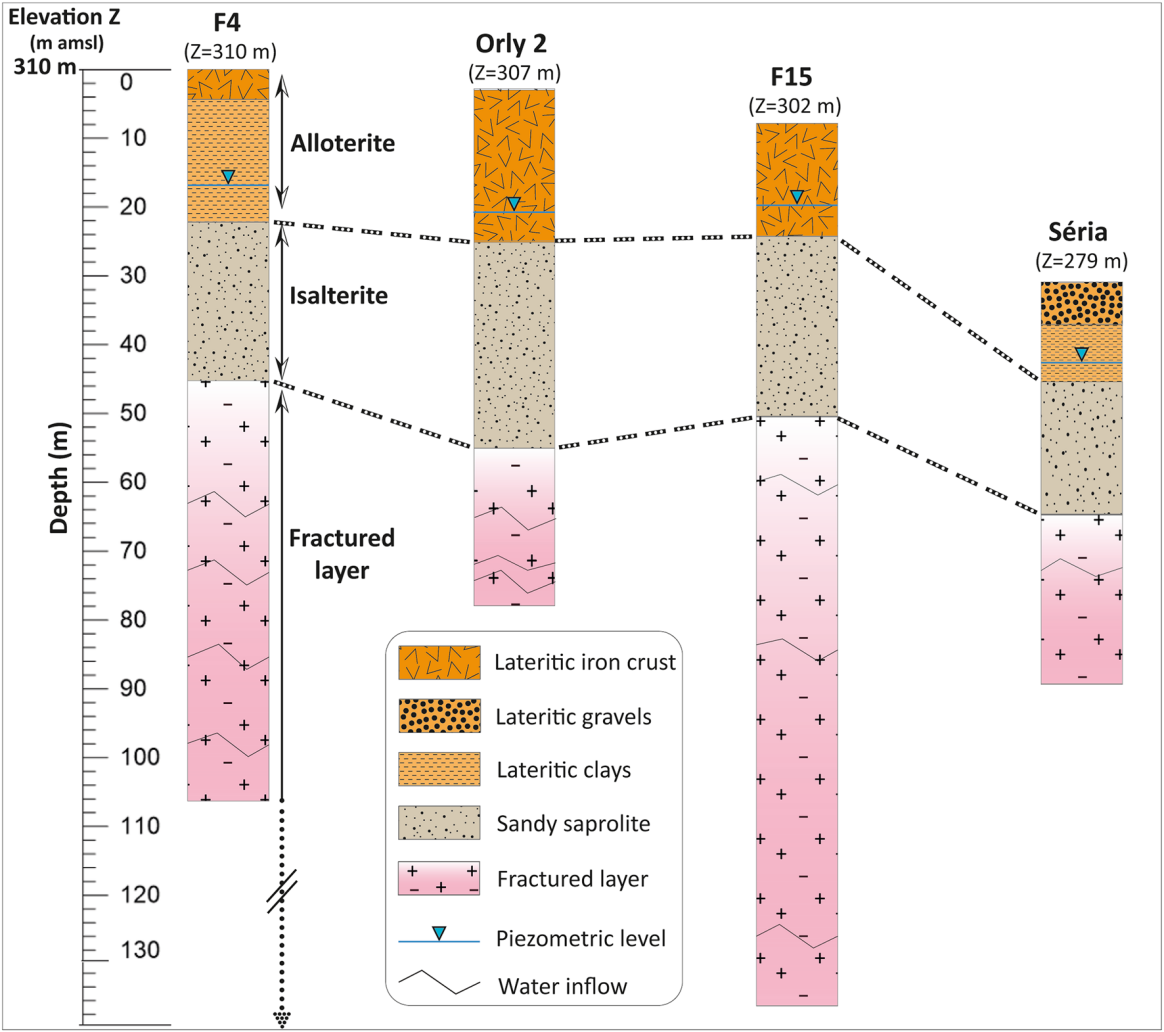
Lithological profiles illustrating the weathering profile at the study site, based on borehole log observations. This graph was produced using Adobe Illustrator V25.1.

Analysis of the lithological cross-sections of the boreholes (observations made during drilling, notably on cuttings) shows the succession of three very distinct horizons within the weathering profile (Fig. 2), which follow a classic vertical structuring^5^ with, from top to bottom: alloterites (10–40 m thick on complete, non-eroded profiles), isalterites (20–30 m thick on complete, non-eroded profiles), which lie on the fractured layer within which water inflows are observed during drilling. From bottom to top, below the soil horizon, the alloterites comprise the lateritic iron duricrust, locally dismantled into lateritic gravels, and lateritic spotted clays (Fig. 2). The alloterites, with their very low permeability, form an aquitard on this site and in general^5^. The isalterites, on the other hand, constitute a "medium" permeability aquifer (about 10^−6^ m/s)^5^, with an effective porosity that can exceed 5%^5^ (from our laboratory porosity analyses carried out using the imbibition method on intact soil samples). This aquifer is generally, and also in Daloa, tapped by traditional large-diameter shallow wells, usually located in valleys where the piezometric level is close to the ground surface. The fractured basement (the fractured layer) underlies the isalterites. In the Daloa region, statistical analysis of linear discharges and water inflow depths^4,16^ from 316 deep boreholes reveals that the first 35–40 m of the fractured layer constitute its most densely fractured, most productive part (See Appendix 7 in the Complementary Materials).

### Calibration and geoelectric signature of the formations constituting the weathering profile

**Figure 3:**
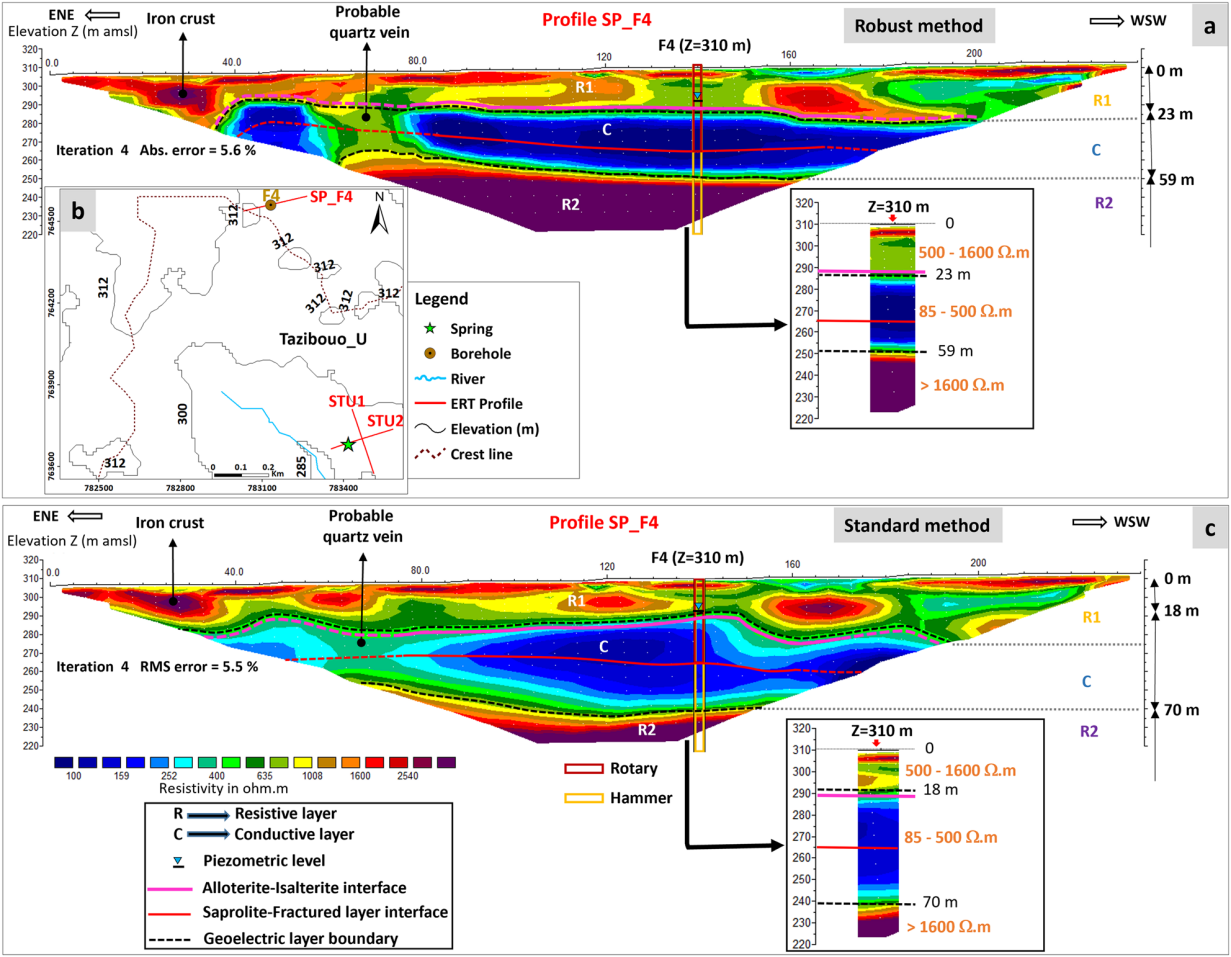
Interpretation of SP_F4 ERT profile with two inversion methods. (**a**): ERT profile inversed with the Robust inversion method; (**b**): location map of SP_F4, STU1 and STU ERT profiles (see the location of this area on Fig. 1); (**c**): ERT profile inversed with the Standard inversion method. Inversions were performed using Res2Dinv V-3.57.36.

The ERT cross sections were inverted with two different inversion methods (“Robust” and “Standard”) which are among the most widely used in geophysics (Fig. 3a, b). All ERT cross sections and inversions show a similar succession of stratiform layers of varying resistivity, with from top to bottom: a resistive layer (R1 = 500–1600 Ω.m) at the surface, up to 20 m thick, followed by a conductive layer (C = 85–500 Ω.m) 30–40 m thick, relatively continuous and homogeneous, and, below, very resistant formations (R2 > 1600 Ω.m).

**Figure 4:**
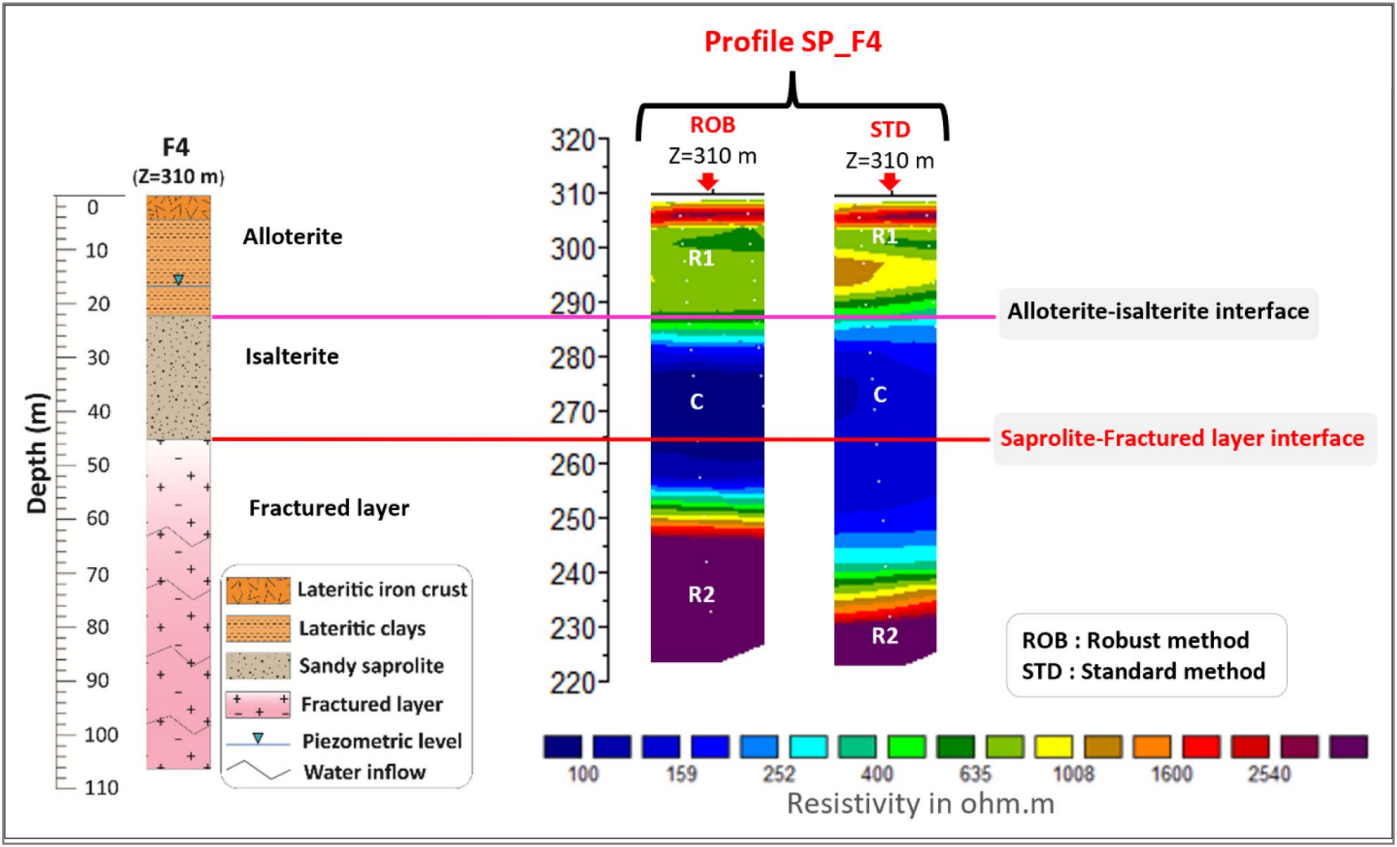
Vertical distribution of resistivity as a function of the inversion method.

Regardless of the inversion method used, comparison of geoelectric profile results with borehole lithology (e.g. borehole F4 in Fig. 4) shows that the resistant geoelectric layer observed at surface corresponds to the alloterites, characterized notably by lateritic formations. This layer is geologically and electrically heterogeneous, and predominantly resistant. Below this resistant layer, a more homogeneous conductive layer appears. This conductive layer corresponds to the lower, water-saturated, part of the saprolite, largely corresponding to the sandy saprolite (isalterite) and to the most superficial part (15–20 m) of the underlying fractured layer. Below this conductive layer, a highly resistant layer follows the conductive layer, via a resistivity gradient that is tighter with the Robust inversion method than with the Standard one. This latter layer corresponds to the middle and lower part of the fractured layer, as the depth of investigation of the ERT profiles does not allow to reach the base of the fractured layer and the underlying unweathered high resistivity rock. The saprolite-fractured layer interface lies at the heart of conductive layer C, frequently at the base of its most conductive part. This "isoline" (base of the most conductive part of conductive layer C) was used to extrapolate this interface; however, the precision of its positioning remains low (accuracy of the order of 5–10 m). The resistivity gradient observed within the fractured layer most likely corresponds to a fracture electrical connectivity threshold (in the sense of the percolation theory). Permeable fractures (identified as water occurrences during drilling) are not visible on the ERT.

Results from both inversion methods show the same sequence of resistive (R1)—conductive (C)—resistive (R2) layers (with R1-C-R2 from top to bottom) (Figs. 3 and 4). The depth and, above all, the progressiveness of the conductive-resistive boundaries (R1-C and C-R2) vary from one inversion method to the other. The upper boundary (R1-C), marked by a relatively abrupt transition, is fairly well defined on the ERT by both inversion methods, although the gradient is slightly stronger with the Standard inversion method than with the Robust one. On the basis of borehole data alone, it is difficult to determine whether this R1-C boundary corresponds to the contact between alloterite and isalterite or to the saturation limit within the weathering profile (piezometric level). However, as this boundary has abrupt variations and locally steep slopes, it cannot correspond to the piezometric level, whose spatial variations are much more regular. The piezometric level is thus not imaged by ERT. The lower limit (C-R2) is not defined in the same way by the two inversion methods. The resistivity gradient is stronger with the Standard method than with the Robust one. On this basis, the Robust inversion method was chosen for the interpretation of all the ERT profiles in this research. Nevertheless, inversions using the Standard method were also taken into consideration to avoid any artifacts associated with inversion.

### Geoelectrical signature of the weathering profile at springs

Given the limited geological information available at the springs, the interpretation of the ERT profiles was mostly based on the calibration of geophysical investigations carried out at the boreholes (see previous section) and on observations made at outcrops, notably at sand quarries located in the saprolite in valleys or close to the springs, or even at the springs themselves.

#### Interpreting ERT profiles

**Figure 5:**
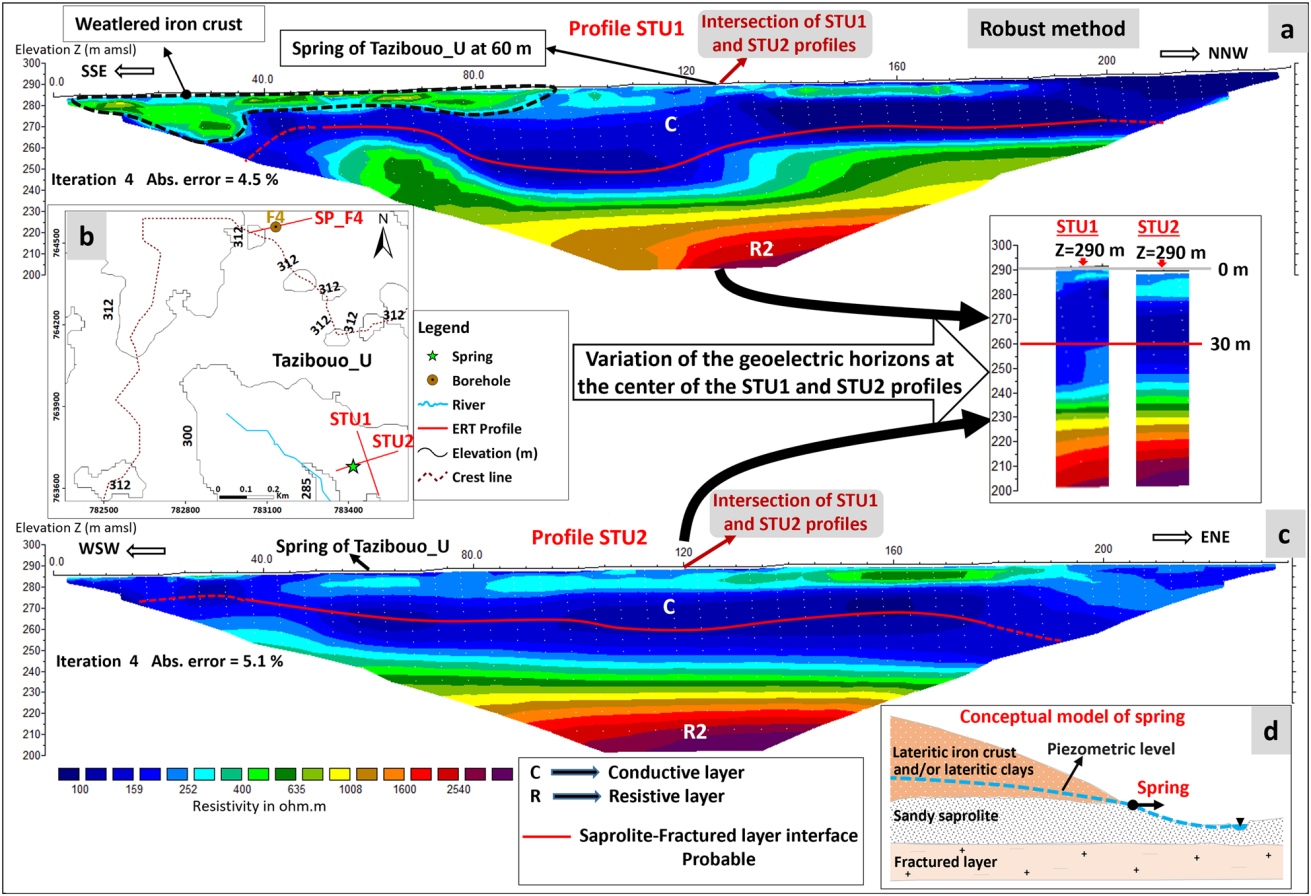
Interpretation of ERT profiles at the Tazibouo_U spring (Robust inversion). (**a**): ERT STU1; (**b**): Location of ERT STU1, STU2 and SP_F4 (see the location of this area on Fig. 1); (**c**): ERT profile STU2; (**d**): Conceptual model of the Tazibouo_U spring.

The STU1 ERT is located 60 m upstream of the Tazibouo_U spring. It was sited perpendicular to the topographical slope and therefore perpendicular to the assumed groundwater flow axis (Fig. 5a). On this profile, the highest topographic heights are found at the highest abscissas (right-hand side of Fig. 5a). The second ERT, STU2, is located perpendicular to the STU1 profile and passes over the spring (Fig. 5b). At their point of intersection (abscissa 125 m on ERT STU1 and abscissa 120 m on ERT STU2), the two profiles show a very similar geoelectric signature and also similar depths for the contacts between the different geoelectric layers. This demonstrates the accuracy of the acquisitions and inversions.

The geoelectric layers observed on the STU1 and STU2 ERT are, from top to bottom, as follows: - a first conductive layer (resistivity 80–500 Ω.m) with a thickness fluctuating between 30 and 55 m is observed near the surface. Observations made during the building of the catchwork of this spring revealed the presence of granitic arenas (isalterites) at a depth of 3 m, topped by a tin sandy and sandy-clayey layer (soil), confirming the geological calibration of ERT carried out on the basis of drilling data (cf. previous section). The laterites and lateritic duricrust observed on the top of the plateau (particularly in borehole F4) would thus have been partially or completely eroded in this valley. Based on the calibration performed on the boreholes, the depth of the transition between the isalterites and the fractured layer would thus be located between 12 and 40 m below the topographic surface along the ERT, at elevations between 275 and 252 m asl. This interface is relatively flat on the STU2 profile. It shows variations of around 20 m in amplitude on the STU1 profile; - the underlying layer correspond to the resistivity gradient observed on the profiles described in the previous section, which images the upper and middle parts of the fractured layer, whose resistivity is between 500 and 1600 Ω.m in its upper part and > 1600 Ω.m beyond that.

The spring thus emerges from the isalterites, which constitutes the aquifer that feeds it (Fig. 5d). The transition between the isalterites and the fractured layer lies several tens meters below the spring.

**Figure 6:**
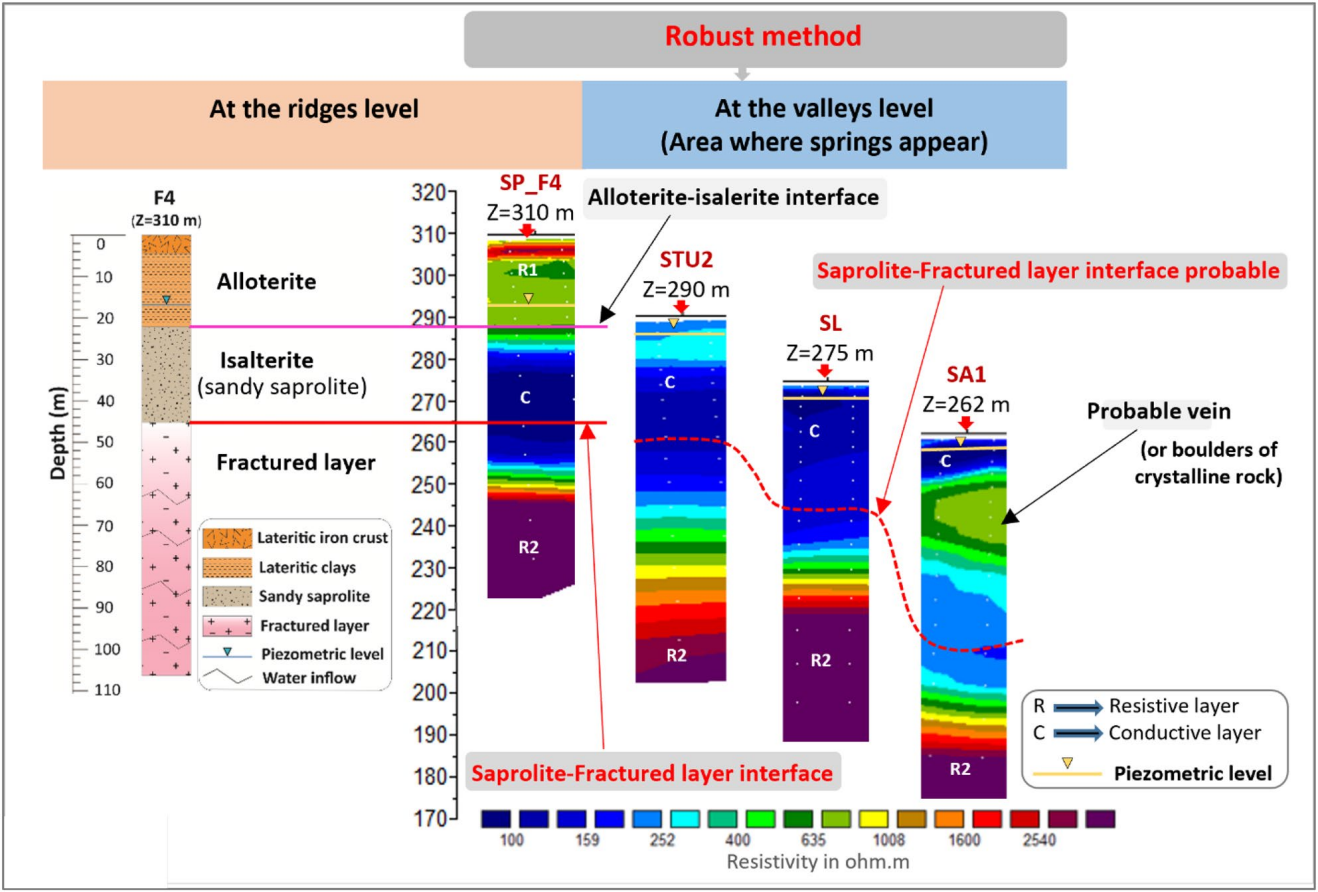
Lateral evolution of geoelectric signatures from the plateau to the valleys. The SL and SA1 ERT are presented in Appendix 4 of Complementary Materials.

Electrical imaging of the SL, SA1, SA2 and SZ profiles (see Appendix 4 and 5 in the Complementary Materials), which are also located on or near springs, show the same geoelectrical succession as the STU ERT, with a conductive medium near the surface, with resistivities between 80 and 500 Ω.m, corresponding to isalterites. Auger sampling at several of these sites confirmed the presence of coarse sand at 1 m depth (below the pedological layer). Beneath this conductive layer, resistivity increases as on the STU profiles and on profiles located at a distance from the springs. The geoelectrical profiles observed on the plateaus and in the valleys, and therefore their different weathering profiles, are therefore similar. The only difference is that weathering profiles in the valleys were partly eroded. Everywhere on this site, springs emerge in the heart of the isalterites (Fig. 6). Unlike springs emerging from sandstones, where springs outflow from conduits located where fractures have been enlarged by weathering and/or erosion due to the groundwater flow^6,7^, at the outlet of the springs in the saprolite (and elsewhere in the saprolite), there are no fractures. Consequently, there are no conduits either.

In detail, we also note, on almost all of these ERT, the presence of intermediate resistivity structures (resistivity: 500–1600 Ω.m) within the subsurface conductive layer (C). These structures are associated with local lower depth or deepening of the base of the conductive layer (C) at the expense of the underlying resistant layer (R2), which corresponds to the median part of the fractured layer (Fig. 6). These vertical variations indicate the presence of heterogeneities within the weathering profile linked to differential weathering of the parent rock: quartz veins less weatherable than their host rock (some were observed in the field), pegmatite or green rocks veins also observed on some drill logs, boulders, etc.

### Typology of springs in a weathered crystalline rock context

**Figure 7:**
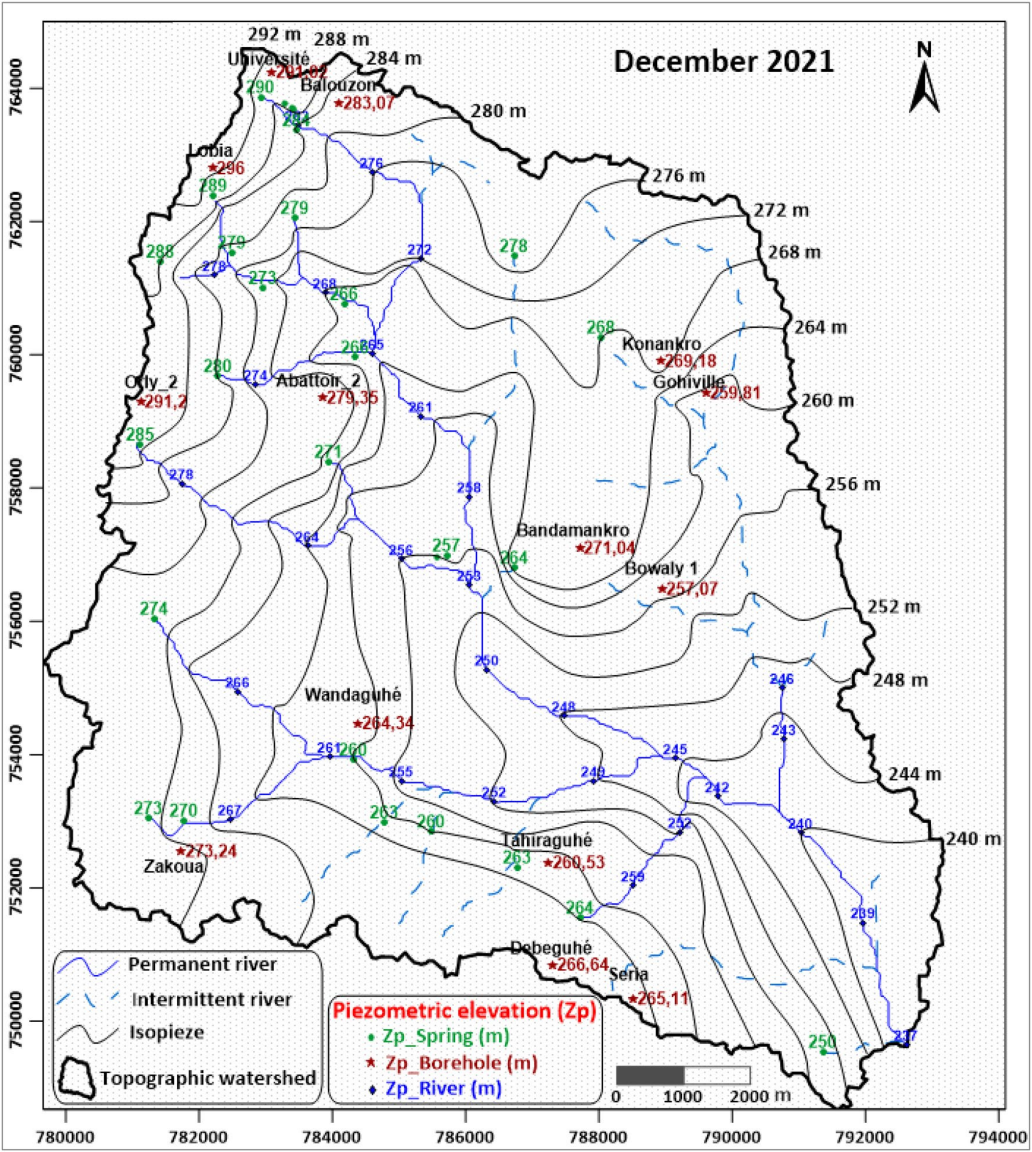
Low-water piezometric map of the Tétégbeu river watershed (Dec. 2021). The Grass Green dots and associated numerical values represent the location and elevation (in m amsl) of groundwater at the springs, respectively. The Ruby Red points and their associated numerical values represent the location and piezometric level of boreholes, respectively. Blue dots and associated numerical values represent the location and water level in leveled points in the river, respectively. Refer to the text for the methodology used for the contouring. This graph was produced using Surfer 19 and was manually improved.

Figure 7 shows that the elevation of springs, of the piezometric level in boreholes and the elevation of the perennial parts of watercourses observed during low-water periods are perfectly consistent each with each other (the same applies to high-water periods, but only when there is no more runoff in the river due to a recent rainfall event). As groundwater supplies the surface water flow, by taking all these data into account to draw up the piezometric map, we were able to obtain a faithful representation of reality (Fig. 7). This approach proved much more accurate than the one using only the piezometric elevation of the 14 boreholes surveyed. The piezometric map (Fig. 7) shows that the groundwater flow follows the topography, as observed in most crystalline aquifers^5^. The perennial parts of the Tétégbeu River drain the aquifer, that is unconfined. The river therefore owes its low-water flow to the groundwater outflow. Springs also drain the aquifer. The piezometric gradient is of the order of 0.5%, which corresponds to a fairly high value, consistent with the modest permeability of the isalterite aquifer.

**Figure 8:**
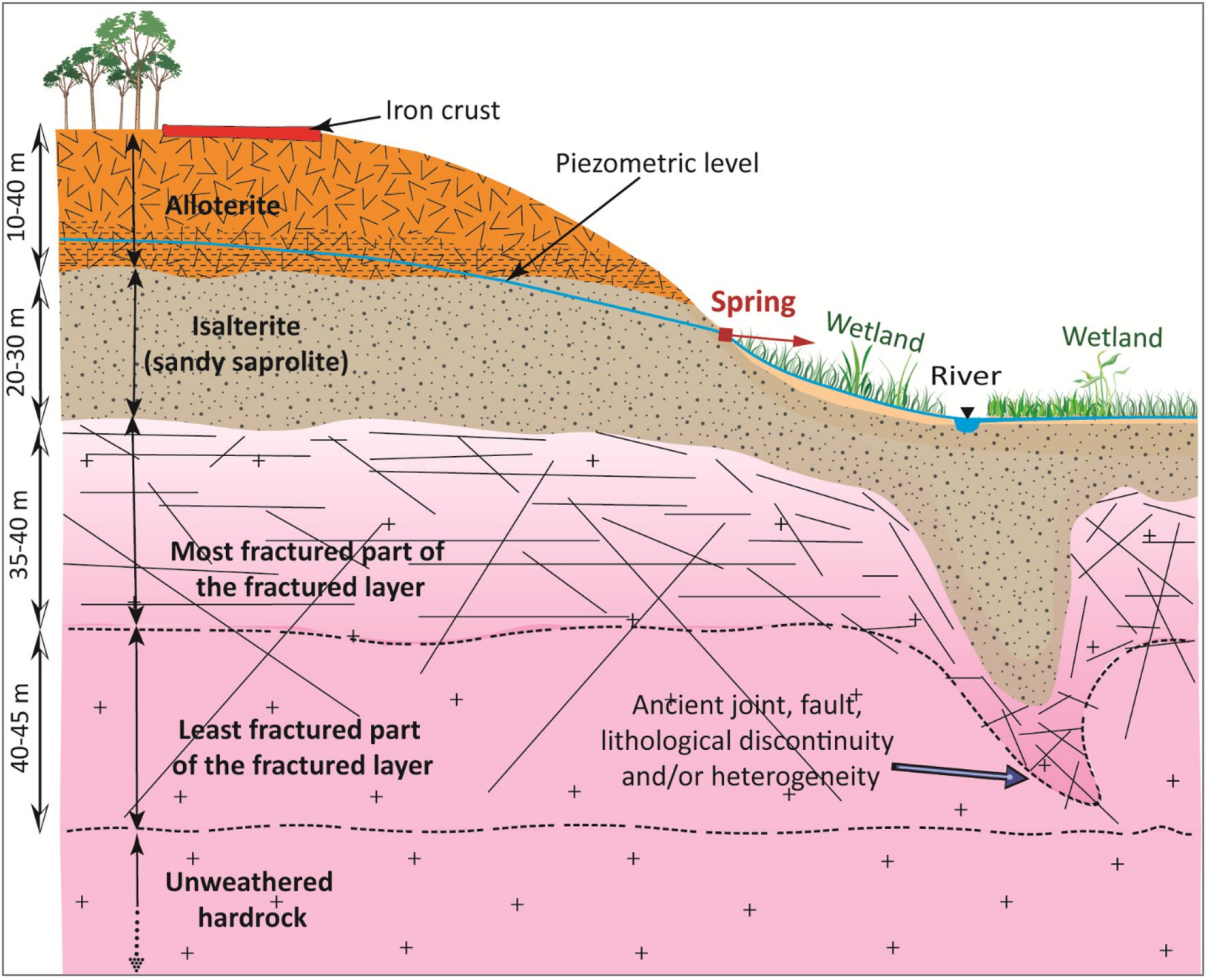
Generalized conceptual model of springs in a weathered-fractured crystalline rock context. This graph was produced using Adobe Illustrator V25.1.

The combined analysis of information from borehole geological cross-sections, ERT, topographic and piezometric maps provides a coherent hydrogeological conceptual model (Fig. 8) of the study area, enabling us to understand the context of spring emergence in a weathered crystalline rock. With regard to the geological structure of the weathering profile, it is shown that the lateritic formations observed on the plateaus (relicts of the weathering paleosurface) have been eroded into the valleys. In the valleys, only isalterites outcrop and, possibly, recent iron crusts of late secondary weathering of this eroded weathering profile. The springs emerge from and are fed by the isalterite aquifer. The fractured layer, usually tapped by boreholes, is located some tens of meters deeper. Springs are tapped in the upstream part of the area, where the piezometric level begins to tangent the topographic surface. Downstream wetlands are also zones of diffuse emergence of the isalterite groundwater.

## Discussion

The combination of results from ERT on the plateaus and in spring zones, with borehole lithological cross-sections and outcrop observations at a weathered-crystalline aquifer site has enabled us to characterize the architecture of the aquifer that supplies the springs. ERT profiles revealed a weathering profile several tens of meters thick, composed of three main geoelectric layers below the plateaus and two geoelectric layers in the spring emergence zones (valleys). These results are in line with those of previous studies carried out in a similar geological environment, but which were not aimed at understanding the context of spring emergence^26,32,42^. From top to bottom, we observe: a resistant layer (500–1600 Ω.m) including locally relatively more conductive zones, then a highly conductive layer (80–500 Ω.m) and a highly resistant layer (resistivity > 1600 Ω.m). The resistant layer observed near the surface on the plateaus doesn’t exist in the valleys, notably near the springs, where isalterite outcrops are also frequently observed. This clearly shows that the laterites and iron crust observed on the plateaus, as well as a part or the totality of the underlying spotted clays have been eroded in the valleys. The slightly more resistant subsurface soils observed are relicts of these iron crust or, more likely, late secondary ones^43,44^. The zones of high resistivity (resistivity > 1600 Ω.m) that stand out within the resistant layer at the surface on the plateaus would correspond to such duricrust observed at the surface at many points, where it is not covered by a gravelly or lateritic layer (duricrust in the process of being dismantled). The wide variations in resistivity of this resistant layer reflect the highly heterogeneous nature of the lateritic formations. This shows that, in these areas of weathered crystalline rock, the alloterite layer comprising these lateritic formations is highly heterogeneous. Based on direct field observations, zones of resistivity above 1600 Ω.m can be attributed to areas where the duricrust is highly indurated and intact, or to the presence of quartz veins within these lateritic formations (See Appendix 6 in the Complementary Materials). Zones of lower resistivity characterize areas where the duricrust has been fractured and cracked by dismantling, forming laterites, or lateritic gravels. Such lower resistivity zones also correspond to lateritic clays^29,45^. This resistant layer and its heterogeneities observed in the subsurface on ERT sited on plateaus do not appear on the ERT sited in the valleys, particularly at or near spring emergence zones, where the underlying conductive layer characterizes isalterites near the topographic surface. This unambiguously shows that the springs emerge from the isalterites, at 20–30 m above its contact with the underlying fractured layer. The saprolite thus constitutes the aquifer supplying the springs, with the upstream emergence zone corresponding to the intersection of the piezometry with the topographic surface. The statistical analysis of linear flow rates and the vertical distribution of water inflows within the fractured layer revealed that the most densely fractured and therefore productive part of the fractured horizon is located in the first 35–40 m below the base of the saprolite. These are emergence springs^46^, the discharge of which depends on the thickness of the aquifer slice tapped. The information gathered during our various field missions reveals that most of these springs are developed by local populations, and generally capture locally a thickness of 2–3 m of the aquifer. This is consistent with the low discharge (0.3–2 L.s^−1^) measured at these springs during our fieldwork (during low water stage). These springs, rarely isolated, are well distributed along the outcropping area of the saprolite aquifer (Fig. 1), at medium elevation in the valleys. It is noticeable that no spring was observed near the main watercourses in the downstream part of the watershed; this is due either to the fact that there are diffuse groundwater outflows along the watercourse and in the wetlands, and/or that the river eroded there the weathering profile below the base of the saprolite. Spatially distributed river discharge measurements and groundwater modeling should answer this question in a further stage of the research. Detailed discharge measurements performed during the 2022 low water stage in the Tazibouo and Balouzon area (Fig. 1) show that the 4 springs of this area have a total discharge of 2 L.s^−1^ whereas the stream, immediately downstream them, has a 4.6 L.s^−1^ discharge (the total discharge of this area being about 6.6 L.s^−1^, which means about 1.6 L.s^−1^.km^−2^; or about 50 mm/year, which gives by the way a minorant of the annual aquifer recharge). These data confirm that the springs only partially drain the aquifer, about two thirds of the groundwater “escaping” them and diffusely emerging in the wetlands and along the watercourse. A more suitable development of the springs, by increasing for instance the thickness of the slice of aquifer tapped, would therefore increase their discharge, but transform them into kind of dugwels, and reduce the stream discharge. This increase of the springs’ discharge would be a maximum of about 4 L.s^−1^. It means that in these areas the potential for spring development is about 1.5 L.s^−1^.km^−2^ (or 50 mm/year). Compared with boreholes, spring exploitation is subject to the disadvantages of reduced discharge during the dry season; moreover, springs buffer inter-annual recharge variations to a lesser extent and does not, as borehole does, allow for peaks in water uses. From a quality point of view, the isalterite aquifer is exposed at the surface; its groundwater is vulnerable. Other parts of this research project will deal with groundwater quality and clarify this statement.

The rare resistant anomalies visible on the ERT in the isalterite are interpreted either as boulders or to the presence of veins. Indeed, numerous quartz blocks, possibly the result of the dismantling of veins, are visible within the lateritic layers (See Appendix 6 in the Complementary Materials). On this site, both outcrop observations and the geoelectric signature rule out the presence of a laminated layer at the base of the saprolite^37^.

## Conclusion

The development of a conceptual hydrogeological model of springs in this research responds to the urgent need to mobilize and effectively manage groundwater resources to support and maintain the supply of drinking water to populations in the crystalline regions of Côte d'Ivoire. This study explored the effectiveness of ERT in characterizing the context of groundwater emergence in weathered crystalline environments. To this end, ERT were sited on the plateaus and in the valleys where springs appear. The geoelectric layers revealed were compared with outcrops and observations performed during boreholes’ drilling. A statistical analysis of the linear discharge of 316 deep boreholes and of the depths of water inflows was also carried out across the Daloa region to determine the thickness of the most productive part of the fractured layer. The main conclusions are as follows:

- ERT is effective in describing the stratigraphic structures and hydrogeological characteristics of the weathering profile. However, ERT cannot image the individual fractures of the fractured layer that were observed during drilling.
- The clayey sand, sand and granitic arenas that constitute the isalterites aquifer supplies the springs. The underlying fractured layer, which top is 20–30 m deeper than the springs level, is not directly implied in this supply.
- Statistical analysis of linear discharge and of the vertical distribution of water inflows beneath the saprolite shows that the most permeable part of the fractured layer is located in the first 35–40 m below the base of the saprolite. This layer is tapped by most borewells.

Although 2D electrical resistivity techniques are effective in identifying weathering profile structures, inclunding zones of intense fracturing (the fractured horizon), they are limited in imaging individual fracture planes and quantifying the volume of mobilizable water in the aquifer. We propose further geophysical investigations using 3D electromagnetic imaging and Proton Magnetic Resonance (PMR) to quantify the volume of water stored in the aquifer, and hydrogeological modeling to quantify the volume of mobilizable groundwater.

## Methods

The methodological approach adopted consisted in describing the hydrogeological structure (weathering profile) of the aquifer system using electrical resistivity tomography (ERT) calibrated on borehole and outcrop data, then characterizing the piezometry.

### Description of weathering profile at borehole scale

The description of the vertical structuring of the weathering profile is based on the interpretation of 316 borehole data sheets with an average depth of 68 m distributed over the Lobo River watershed, including 16 in the study area (some of which are shown in Fig. 2). Drilling data sheets were collected from the Human Hydraulics Department (DHH), based in Daloa. These drilling data sheets are similar to those described in details by Aoulou et al.^4^. Interpretation of the lithologies was enriched by the observation of fractured and weathered crystalline rock outcrops and sand quarries.

### Fractured layer thickness

The method implemented is based on recent concepts of permeability linked to weathering in crystalline aquifers and the method proposed by Courtois et al^16^ and adopted by Aoulou et al.^4^ in a geological environment similar to the study site. One of the main results of the method was the characterization (thickness, productivity) of the fractured layer in crystalline aquifers. Productivity is expressed in terms of linear discharge (m^3^/h/m) and its variation was observed as a function of drilling depth beneath the base of the saprolite. On the basis of the conceptual model of hard rock aquifers, Courtois et al.^16^ defined a new parameter that they named "linear discharge " (Eq. 1):

1 $$\begin{array}{c}{\text{q}}_{\text{i}}\left({\text{l}}_{\text{i}}\right)=\frac{{\text{Q}}_{\text{i}}}{{\text{l}}_{\text{i}}}, i=1,n\end{array}$$

where Q_i_ is the instantaneous discharge (m^3^/h) of borehole number i; l_i_ is the length of the borehole i below the base of the saprolite (m); q_i_ (l_i_) is the resulting linear discharge of the borehole i (m^3^/h/m) and n is the number of borehole considered.

The cumulative percentage of linear discharge under the saprolite base was determined and plotted as a cumulative curve from Eq. (2):

2 $$\begin{array}{c}{\text{p}}_{\text{q}}\left(\text{L}\right)=\sum_{{\text{l}=\text{L}}_{\text{min}}}^{\text{l}=\text{L}}{\text{q}}_{\text{i}}\left(\text{l}\right)/\sum_{{\text{l}=\text{L}}_{\text{min}}}^{{\text{l}=\text{L}}_{\text{max}}}{\text{q}}_{\text{i}}(\text{l})\end{array}$$

where q_i_ (l) is the linear discharge of borehole i calculated with borehole length l under the saprolite base (m^3^/h/m) and p_q_ (L) is the cumulative percentage of linear discharge (%) calculated for the sample composed of boreholes whose length under the saprolite base is less than L (m). The cumulative percentage p_q_ (L) is calculated for all possible values of L present in the borehole dataset, ranging from L_min_ to L_max_. By definition, the cumulative percentage ranges from p_q_ (L_min_) = 0% to p_q_ (L_max_) = 100%.

A graphical representation of cumulative linear discharge as a function of borehole length l below the base of the saprolite has been produced as proposed by Courtois et al.^16^. Within the cumulative linear discharge curve, a median part of the curve with a linear trend is generally distinguished, with a relatively gentle slope, corresponding to the highest linear discharge, which characterizes the thickness and productivity of the most productive part of the fractured layer. Beyond this point, linear discharges decrease and become zero as the base of the fractured layer is passed. The latter part of the curve characterizes the deepest part of the fractured layer and any underlying permeable discontinuities. The first part of the curve is sometimes steeper at the beginning. This is due to uncertainty as to the location of the saprolite base. The average productivity of the most transmissive part of the fractured layer can be quantified by Eqs. (3) and (4):

3 $$\begin{array}{c}{\text{q}}_{\text{M}}\left({\text{L}}_{\text{u}}\right)=\sum_{\text{l}=\text{Min }\left({\text{l}}_{\text{i}},\text{ i}=1,\text{ n}\right)}^{{\text{L}}_{\text{u}}}{\text{q}}_{\text{i}} \left(\text{l}\right)/{\text{j }(\text{L}}_{\text{u}}),\text{ i}=1,\text{ n}\end{array}$$

4 $$\begin{array}{c}{\text{Q}}_{\text{M}}\left({\text{L}}_{\text{u}}\right)={\text{q}}_{\text{M}}\left({\text{L}}_{\text{u}}\right)\times {\text{L}}_{\text{u}}\end{array}$$

where L_u_ (m) is the "useful thickness or thickness of the most productive part" of the fractured layer (defined either by the slope method or the percentile method); q_M_ (L_u_) is the average linear discharge (m^3^/h/m) evaluated for the "useful thickness (L_u_)"; j (L_u_) is the corresponding number of boreholes; and Q_M_ (L_u_) is the resulting average discharge (m^3^/h) for "theoretical" boreholes that would intersect the entire "useful thickness" (length L_u_) of the fractured layer. In the case of this study, L_u_ was determined using the slope method as recommended by Aoulou et al.^4^, who have worked in geological environments similar to the study site.

The database contains information on the depth of water occurrences observed during drilling. Water inflows are observed exclusively within the fractured layer (below the base of the saprolite). They are linked to the existence of permeable fractures within the fractured layer. The database contains 316 boreholes well distributed over the Lobo River watershed, with a total of 593 water inflows observed. Firstly, the depth of each water inflow below the base of the saprolite was calculated as the difference between the depth of the water inflow in question and the depth of the base of the saprolite in the borehole in question. Next, the density of water inflows below the saprolite base was estimated using the R-4.2.1 software by the kernel method^47^, which is a generalization of the histogram estimation method. Kernel estimation is a non-parametric method for estimating the probability density of a random variable. The estimated water density follows a Gaussian distribution with parameters (mean = 23.64 m; standard deviation = 17.22 m). Finally, the joint analysis of the distribution of water inflows and the linear discharge curve enabled us to determine the most productive part of the fractured layer. The results obtained are presented in Appendix 7 in Complementary Materials.

### Description of weathering profile with ERT

The methodological approach consisted in creating electrical panels at ridge level (on boreholes F4 and F15, known as "test boreholes") to describe the weathering profile of plateau zones. Next, electrical panels were installed at the edge of the valleys (preferential groundwater zones) to describe the weathering profile of the spring zones. Finally, a comparative analysis of the geoelectric signatures was carried out to deduce the geological formations at the origin of the springs. 2D electrical resistivity tomography (ERT) was used to describe the weathering profiles. The implementation of this technique is divided into two main stages: field investigations and data processing (inversion, calibration and classification).

#### Field investigations

The most commonly used arrays for 2D resistivity measurements are the Wenner, Wenner–Schlumberger, pole-pole, dipole–dipole and pole-dipole arrays^48^. In this research, the electrode configuration chosen was the pole-dipole, with 48 electrodes spaced 5 m apart. This choice stemmed from the strong urbanization of the study area, limiting the space available to extend electrical cables due to roads, buildings and buried electrical cables. For this reason, the choice fell on the pole-dipole array, known for its ability to differentiate complex geological structures at great depth with short ERT cables, as highlighted notably by Kumar et al.^49^. In addition, the pole-dipole array has a significantly higher signal strength than other arrays to obtain high-resolution 2D resistivity data, as well as higher vertical sensitivity and greater depth of investigation on short linear arrays, as confirmed notably by Kumar et al.^49^ and Olivier et al.^32^ who worked in similar geological environments. At site level, two ERT profiles (SP_F4 and SP_F15) were carried out at the plateau level to characterize the geoelectrical succession in the vicinity of boreholes F4 and F15. F4 and F15 are two boreholes in the University of Daloa's water catchment area, designed to provide the campus with a self-sufficient supply of drinking water. Six other ERT, namely SA1, SA2, SZ, STU1 and STU2, passing close to the Labia, Abattoir 2, Abattoir 2 SUD B, Zaragoua and Tazibouo-U springs respectively, were carried out in the dry season, during the low-water period, to characterize the weathering profile in the areas where the springs emerge. The aim of the ERT carried out at plateau level is to ensure that all stratiform geological formations in the weathering profile encountered in the area are intersected. This makes it possible to assess the geophysical signature describing the different lithological formations encountered in these regions, and to make a comparison between the geophysical signature of the ERT made near the test boreholes and that of the ERT made in the areas where the springs emerge. This technique made it possible to calibrate the model using a set of inversion parameter adjustments, while searching for the model that best describes the lithology of the test boreholes.

ERT were acquired using a Syscal-Pro Switch48 resistivity meter (Iris Instruments, France) with 48 metal electrodes regularly spaced 5 m apart over 235 m. The 5-m spacing between electrodes was chosen to ensure both adequate resolution of subsurface resistivity at shallow depths and a maximum depth of investigation of around 90 m. Electrode contact resistance is generally less than 3 kΩ for all profiles. Acquisition parameters for all profiles and different configurations were as follows: acquisition time 30 min (1181 measurements in direct mode and 1181 measurements in reverse mode); Q < 1% (where Q is the standard deviation on resistance measurements, a repeatability characteristic) and injection voltage of 400 Volt. A high Q factor is generally an indication of a low signal-to-noise ratio. The pole-dipole protocol, with a remote injection electrode, maximizes the measurements signal-to-noise ratioand increases the depth of investigation. The position of the remote electrode was determined in accordance with the recommendations proposed by Razafindratsima and Lataste^50^ to minimize its influence on resistivity measurements, with a minimum distance from the remote electrode of 1000 m (perpendicular to the ERT profile).

#### Data processing: inversion, calibration and classification

After acquisition, measurements were filtered to eliminate anomalous values. Raw data from electrical measurements (apparent resistivity) were filtered and processed (correction of inconsistencies,) with Prosys II software (Iris Instruments) on the following criteria: V (signal) > 0.3 mV and Q < 1%.

Data processing includes data inversion, calibration and classification. Data inversion consists in obtaining iteratively interpreted resistivities from the apparent resistivities measured in the field. The inversion routine generally used is based on smoothing using the least squares constraint^51^. The objective function below (Eq. (5)) constrains the inversion in such a way that spatial variations in the calculated resistivities are smoothed.

5 $$\begin{array}{*{20}c} {f = (\uprho_{{{\text{cal}}}} - \uprho_{{{\text{mes}}}} )^{2} + \lambda \left( {\frac{{{\text{d}}\uprho }}{{{\text{dx}}}} + {\text{Z}}\frac{{{\text{d}}\uprho }}{{{\text{dy}}}}} \right)} \\ \end{array}$$

With ρ_cal_: calculated resistivity, ρ_mes_: measured resistivity; ρ: resistivity of the reference model. The parameters λ and Z are, respectively, the damping factor used to define the intensity of the "smoothing" stress in the inversion and the parameter controlling the anisotropy.

The quality of the inversion is monitored by the RMS (Root Mean Squared)^48,51^. RMS measures the sum of the square of the differences between measured and calculated apparent electrical resistivities. The Root Mean Square (RMS) percentage is used to evaluate inversion performance^31^. RMS is defined by the following Eq. (6):

6 $$\begin{array}{c}RMS=\sqrt[100]{\frac{1}{N}\sum_{1}^{N}{\left[\frac{{{(\text{h}}_{0})}_{i}-{{(\text{h}}_{m})}_{i}}{{{(h}_{0})}_{i}}\right]}^{2}}\end{array}$$

where h_0_ is the observed depth (i.e. the true model) of the soil layer at location i, h_m_ is the model (i.e. the inverted model) and N is the number of observations (the total number of measured data sets).

If RMS is equal to 0, it is clear that the results simulated by the model correspond perfectly to the observations; if RMS is greater than 100%, then the model is no better than a predictor using zero^31,52^.

Electrical resistivity inversions were performed using Res2Dinv V-3.57.36 software. The aim of the inversion process is to minimize the objective function, which is the root-mean-square (RMS) error between the modeled and measured resistivities. Minimization is achieved by an iterative process in which the objective function must decrease. Convergence of the minimization is a mandatory criterion. This is because there is an infinite number of models available corresponding to measurements with the same RMS error^48,49,53^. It is therefore important to reduce the number of mathematical solutions to models that are more geologically realistic, using a so-called inversion procedure and incorporating all available prior information into the inversion process. Caterina et al.^54^ recommend adding prior information such as structural constraints, which seems to be particularly appropriate when the boundary is located in areas that are not very sensitive to ERT. However, such prior information is generally absent, and it is obvious that there is no specific set of parameters giving the "real" 2D resistivity distribution. It is therefore proposed to perform various inversions with different methods and specific constraints, so that each inversion imagery is particularly suited to determining the lithological and structural characteristics of the hydrogeological model^37,48,55–57^. Following this recommendation, we used two inversion methods for comparison: robust inversion, or L1-norm, and standard least-squares constraint inversion, or L2-norm^58^. In this study, we refer to robust inversion (L1-norm) as "robust inversion methods" and standard least-squares constraint inversion (L2-norm) as "standard inversion methods". The residual values of the models for L1-norm and L2-norm inversion are expressed as absolute errors (Abs) and root mean square errors (RMS) respectively^58^. According to Marescot^48^, the robust inversion method (ROB) is well suited to environments with strong resistivity contrasts. It minimizes absolute error and provides clearer boundaries between varied layers. ROB inversion provides more accurate results for determining the boundary between the weathering profile and the unweathered hard rock. The standard inversion method (STD) minimizes squared error and is a smoothed inversion. Combined with an anisotropy factor (flatness ratios) of 0.3, this inversion reinforces the horizontal continuity of the geoelectric layers. In this study, each selected inversion method (Robust or Standard) is used to identify and characterize both vertical resistivity anomalies and recognize subhorizontal resistivity contrasts related to the stratiform weathering profile, in both cases using an anisotropy factor of 1. The structures thus revealed with each inversion method are compared with the lithology of the test boreholes. The resistivity values and geometries are then compared with the geological facies of the test boreholes to confirm the reality of the structures, particularly those that best describe the lithology of these boreholes. The resistivity patterns obtained after inversion were classified according to the resistivity ranges corresponding to each geological formation observed on the geological model of the test boreholes. For each spring ERT, resistivity ranges are established and compared with the resistivities and lithology of the test boreholes, before proceeding with the geological and hydrogeological interpretation.

### Piezometric mapping

Groundwater piezometric levels were measured using a probe whose sensor is reactive to the electrical conductivity of the water. When the probe reaches the level of the air/water interface, electrical contact is established between two metal rods, triggering a sound and light signal. The measurements were carried out in December 2021 (low-water period) on 14 boreholes. The boreholes monitored are little disturbed by domestic water abstraction, so the piezometric levels are not significantly influenced. The first step after the measurement campaign was to determine the piezometric values for each borehole surveyed. The piezometric values were determined using Eq. (7). The piezometric map was produced using the kriging method in Surfer 19, integrating the elevations of perennial springs (28 springs), permanent watercourses (210 points digitalized along the perennial part of watercourses) and the piezometric level measured on the 14 boreholes. Elevations were obtained from the Digital Elevation Model (DEM) extracted from STRM (Shuttle Radar Topographic Mission), with a XY resolution of 30 m. It is important to note that the survey of perennial springs and permanent rivers were carried out between December 2021 and February 2022, during the low-flow period. In this study, the type of kriging used is “point kriging” using the “ordinary kriging method”, as it is the most frequently used by many authors due to its ability to give accurate results^59,60^. Interpolation was carried out using the “standard ordinary kriging” option in Surfer. Prediction efficiency was calculated from cross-validation for the entire dataset. Analysis of the cross-validation parameters indicates a Root Mean Square (RMS) of 1.13 m, a variance of 1.28 m, a standard deviation of 1.13 m and a mean of 0.17 m. These accuracy characteristics prove to be satisfactory for good interpolation^61^. Although the cross-validation parameters are satisfactory, the interpolation is an estimate of point values, not exact values at all points. As a result, the value extrapolated from one point to another is not necessarily identical to the value that would be found if a well were drilled at that point. Moreover, interpolations at the edges of the map have a greater uncertainty than those at the center, as there are fewer field data around them. On the other hand, the accuracy is better in the center of the map. Then, on that basis, we manually adjusted the piezometric contours, using the Spline Polyline tool in Surfer, to account for the relationships between the piezometry and the topography that was not considered during kriging.

7 $$\begin{array}{c}H=Z-\left(\text{P}-{\text{H}}_{\text{m}}\right)\end{array}$$

With: Η: Piezometric elevation (m); Ζ: Ground elevation or elevation of natural terrain (m); Ρ: Measured depth (m); Η_m_: well curb height.

### Supplementary Information

Supplementary Information.

## Data Availability

The data used in the study are available on request from the first author.
