# Supplementary material for: Identifying the origin of springs in weathered-fractured crystalline aquifers using a hydrogeophysical approach
Source: Sci Rep. 2024 Jun 5;14:12977. doi: 10.1038/s41598-024-63748-8 (PMC11153216; doi:10.1038/s41598-024-63748-8)
Supplement: Supplementary file 1 — Supplementary Information. [file 41598_2024_63748_MOESM1_ESM.pdf]

scientific reports

Identifying the origin of springs in weathered-fractured crystalline aquifers using a hydrogeophysical approach

Kouassi Jean-Michel Kouassi[1,2]*, Patrick Lachassagne[2], Oi Mangoua Jules Mangoua[1], Abé Parfait Sombo[1] and Brou Dibi[1]

[1] Laboratory of Environmental Sciences and Technologies, Univ. Jean Lorougnon Guédé, Daloa, Ivory Coast

[2] HSM, Univ. Montpellier, CNRS, IRD, IMT Mines Alès, Montpellier, France

* Corresponding author: kouassimichel48@gmail.com, Tel: +33 623568225 / +225 0748365414

Complementary Materials

Appendix

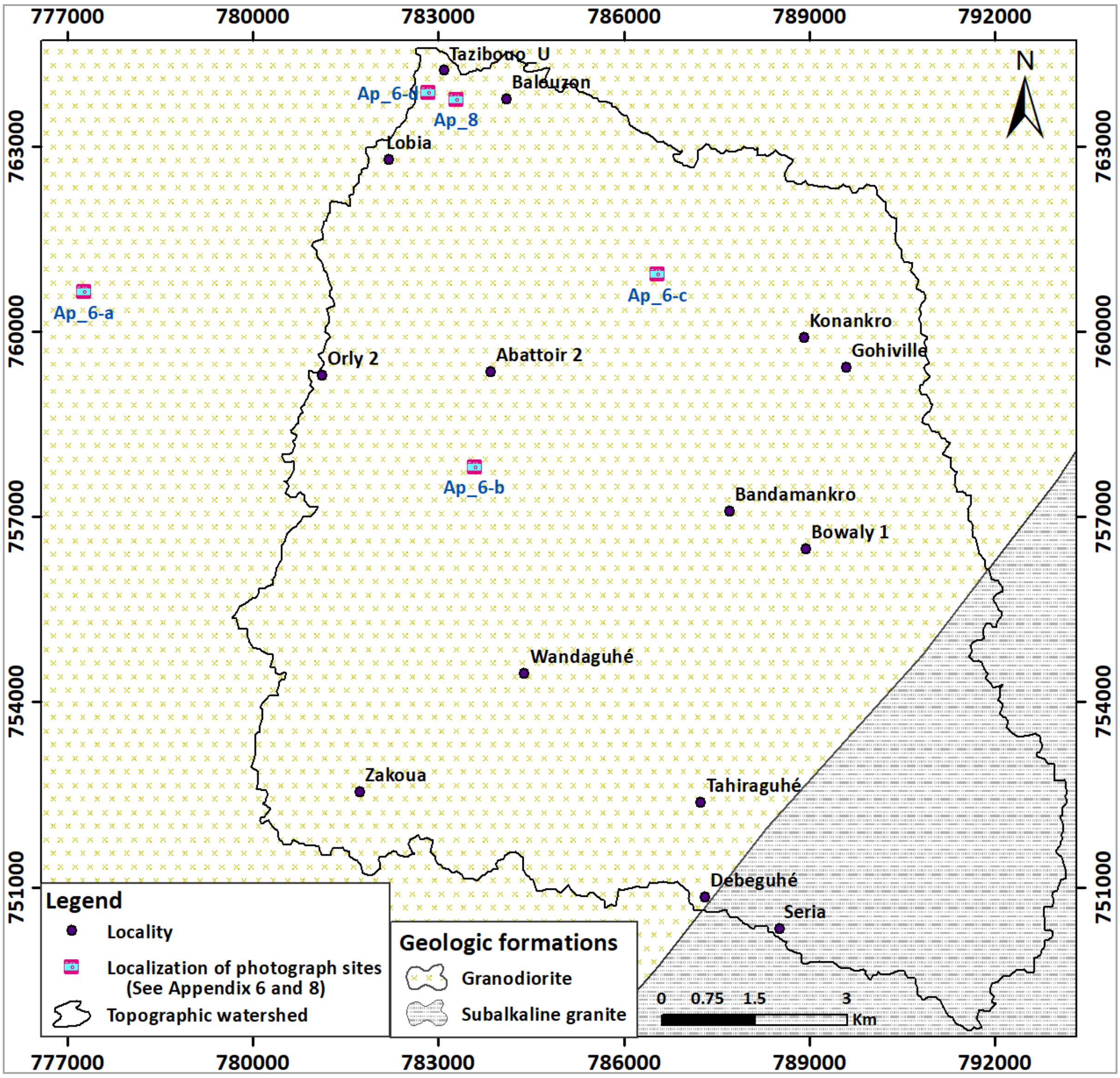

Appendix 1: Geological map of the Tétégbeu River watershed (Delor et al., 1995). This graph was produced with ArcGIS 10.2 software.

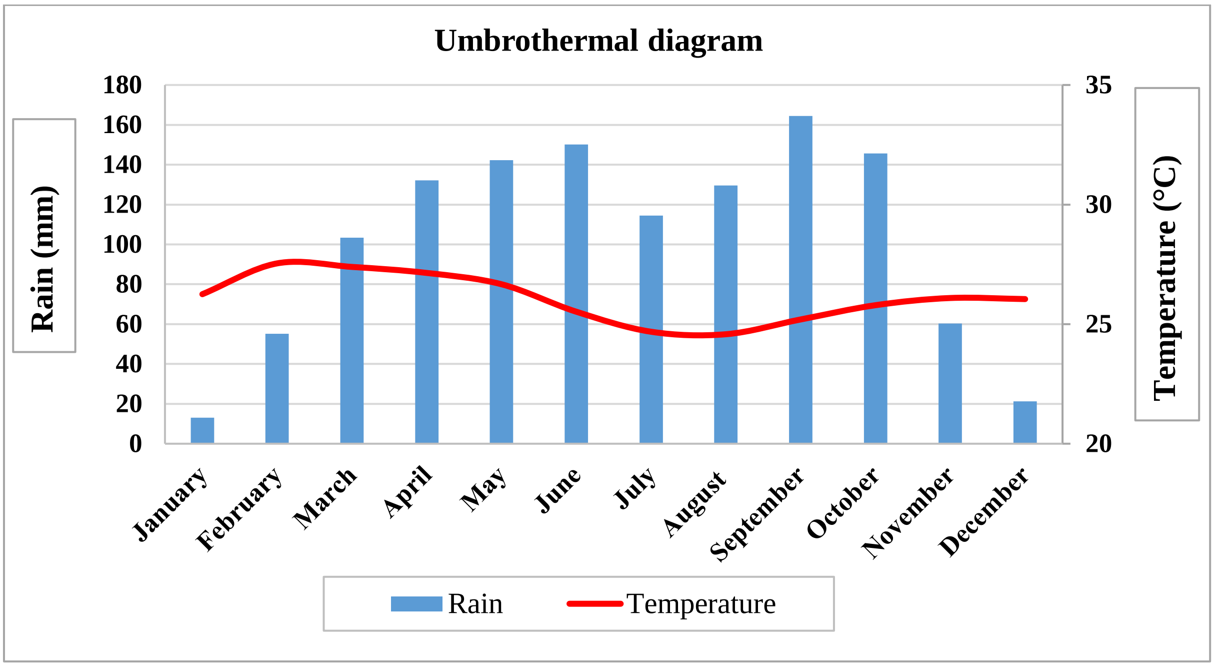

Appendix 2: Monthly average of rainfall and air temperature at the Daloa station (1990-2017).

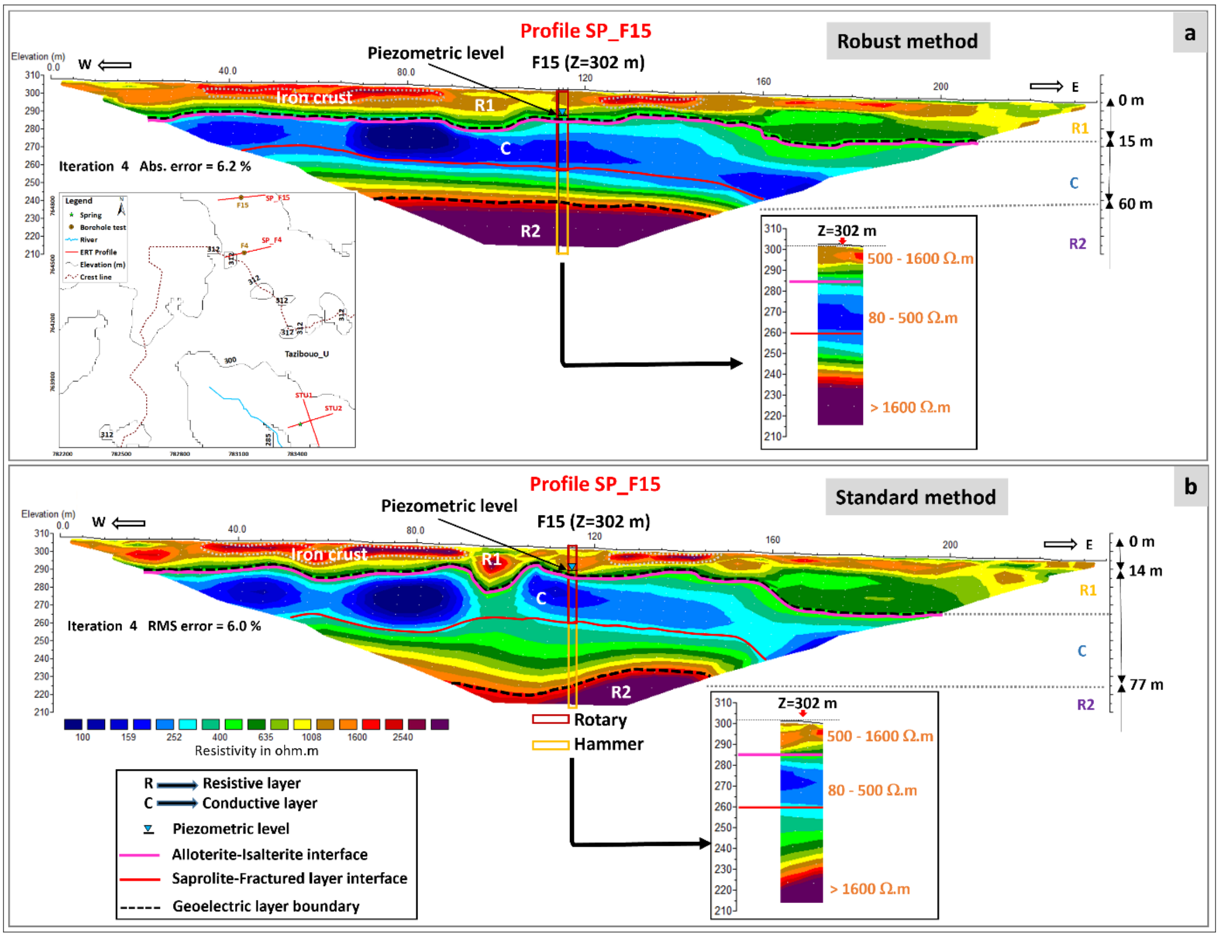

Appendix 3: Interpretation of the SP_F15 ERT. **a**: Robust inversion method; **b**: Standard inversion method.

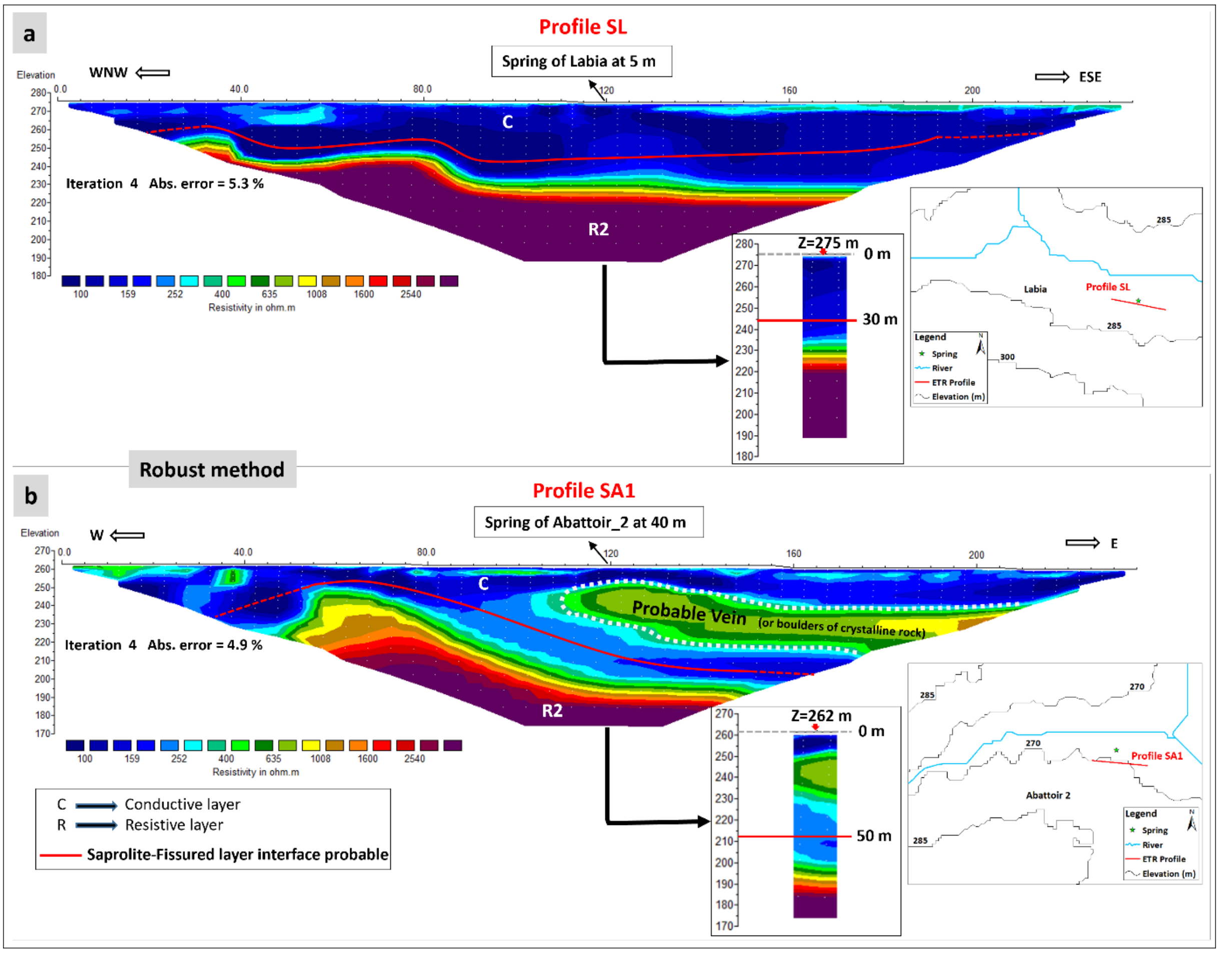

Appendix 4: Interpretation ERT the sources: **a**: profile SL; **b**: profile SA1.

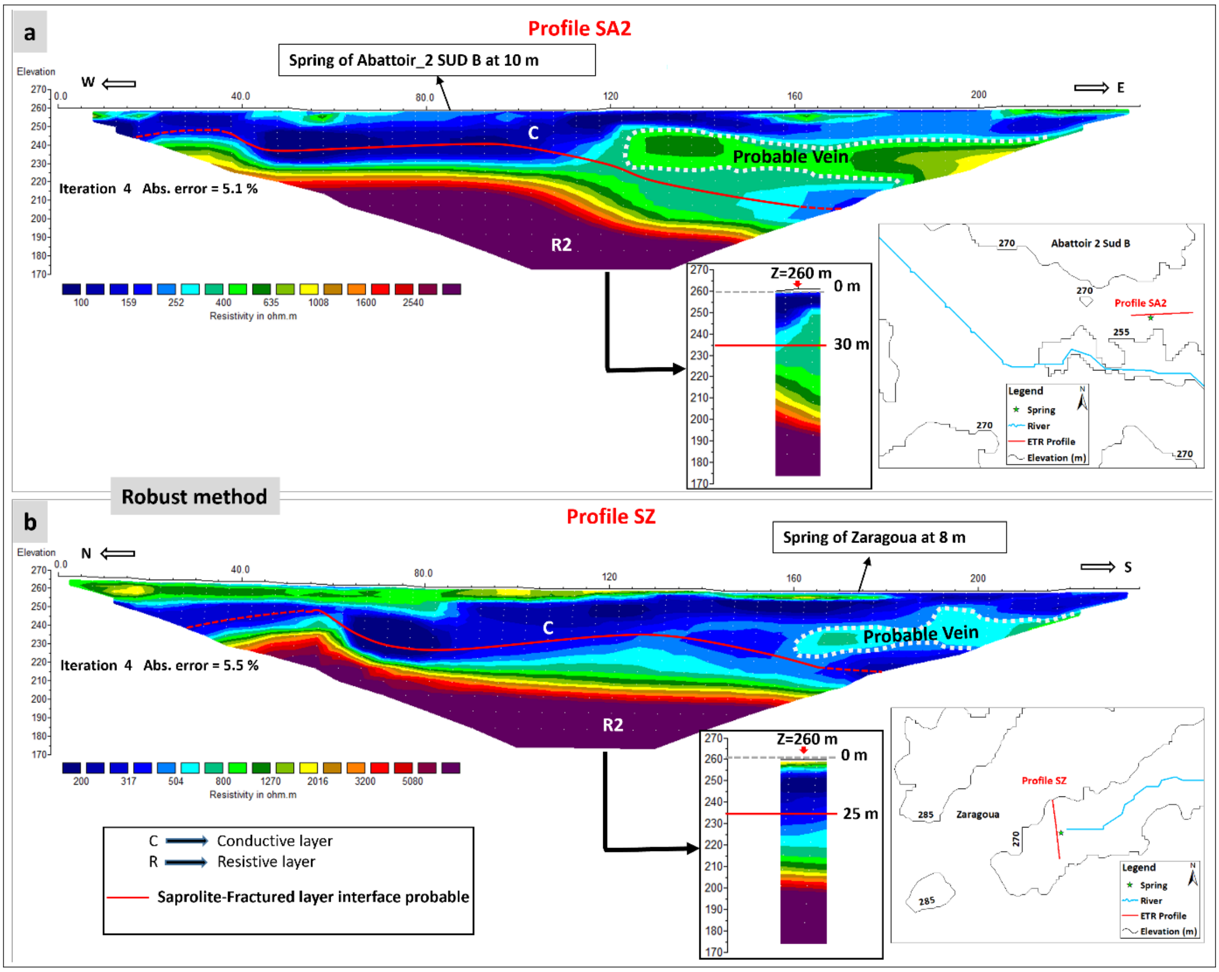

Appendix 5: Interpretation ERT the springs: **a**: profile SA2; **b**: profile SZ.

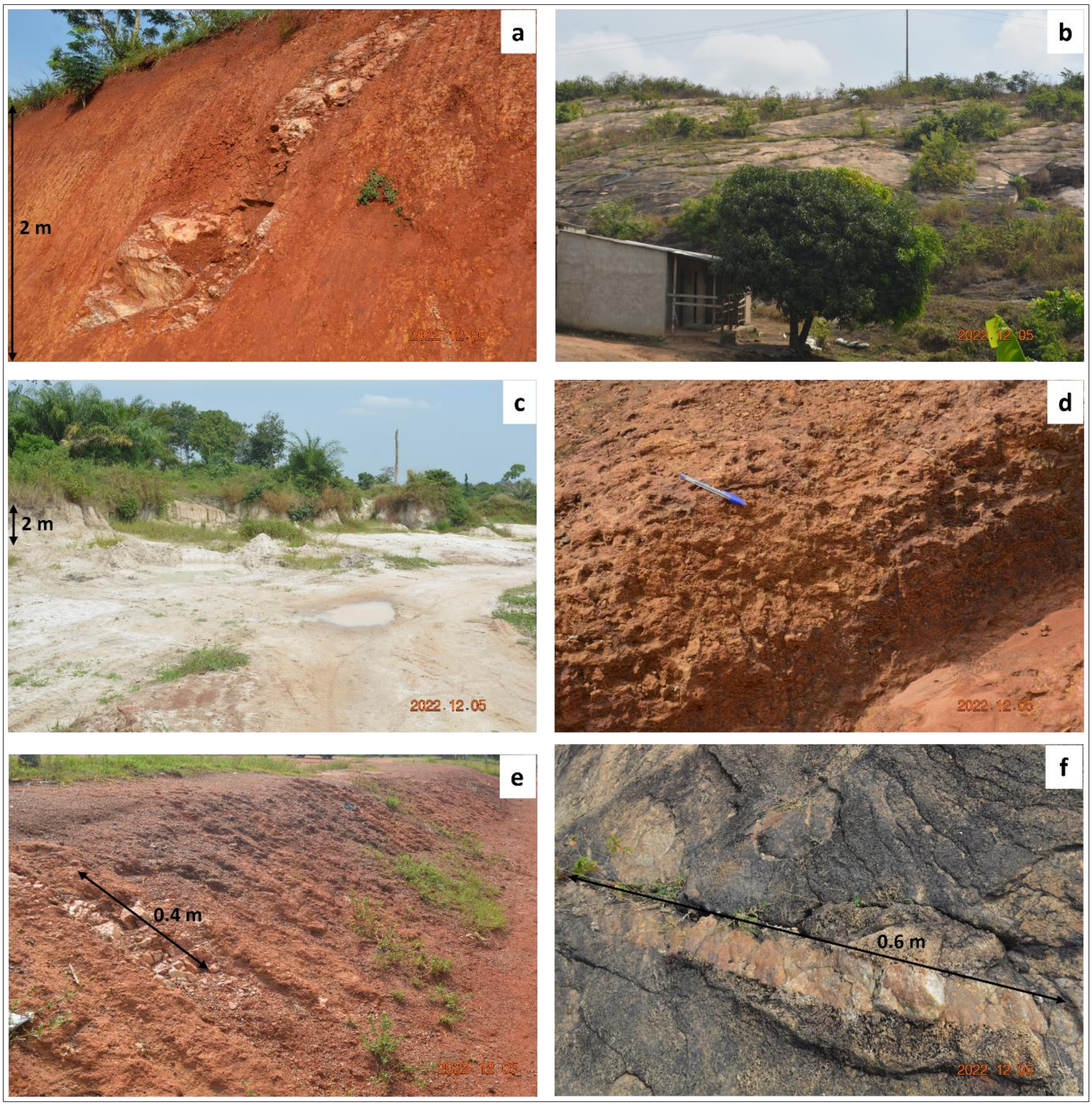

Appendix 6: Photograph showing some of the geological formations observed in the study site (location on Appendix 1). **a**: Quartz vein in to lateritic formations; **b**: Outcrop of the fractured layer in granodiorite; **c**: The aquifer (isalterite) at the origin at the springs; **d**: iron crust; **e**: Quartz vein surface signatures (in saprolite); **f**: Quartz vein surface signatures (in crystalline rock).

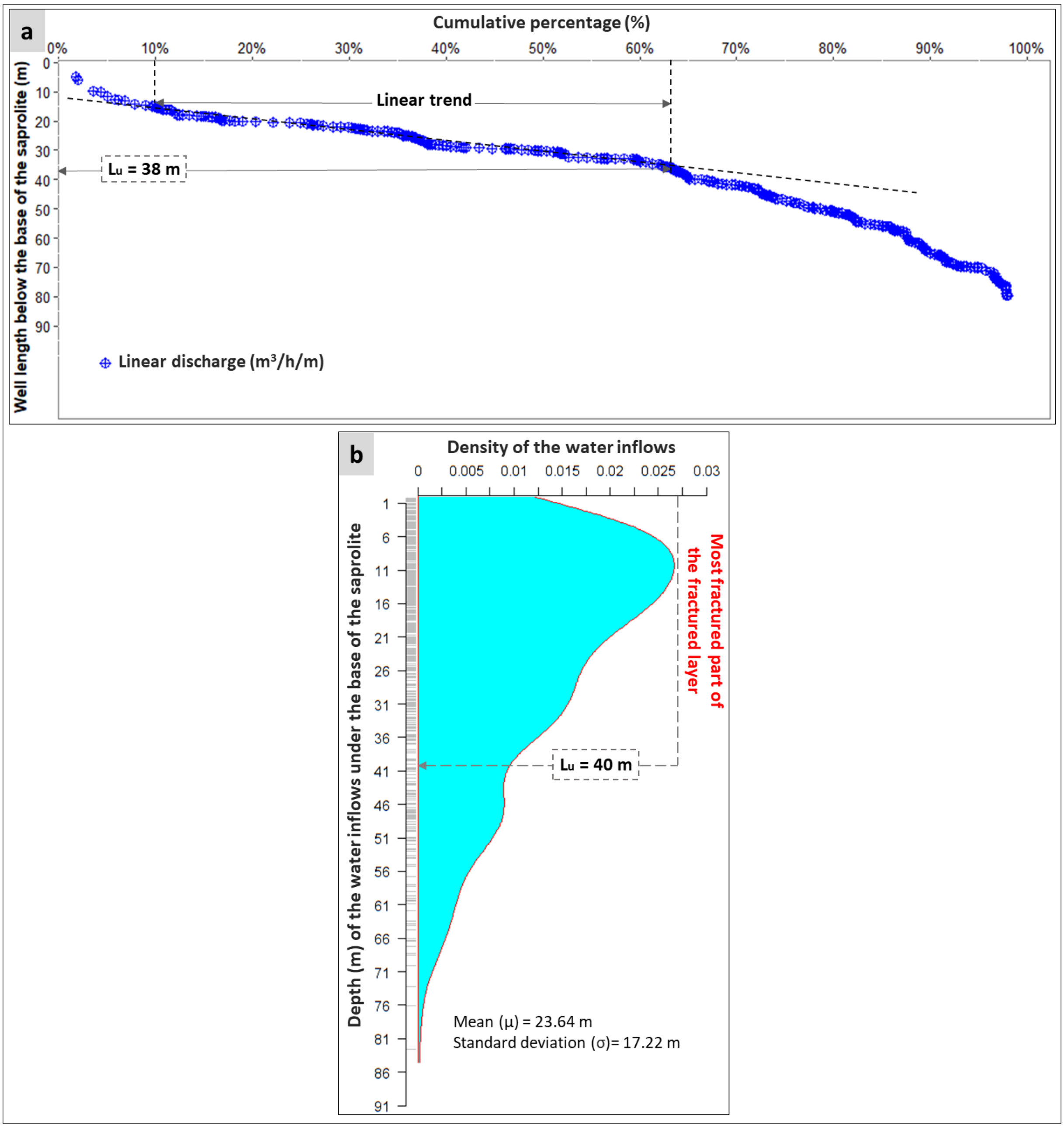

Appendix 7: Mapping of the thickness of the most fractured part of the fractured layer. **a**: Cumulative percent linear discharge to estimate the thickness (Lu) of the most densely fractured part of the fractured layer. **b**: Water inflows density curve using the kernel method to characterize the distribution of hydraulically active fractures within the fractured layer. Lu (m) refers to the "effective thickness or the most productive or densely fractured part" of the fractured layer. These graphs were produced with the R-4.2.1 software.

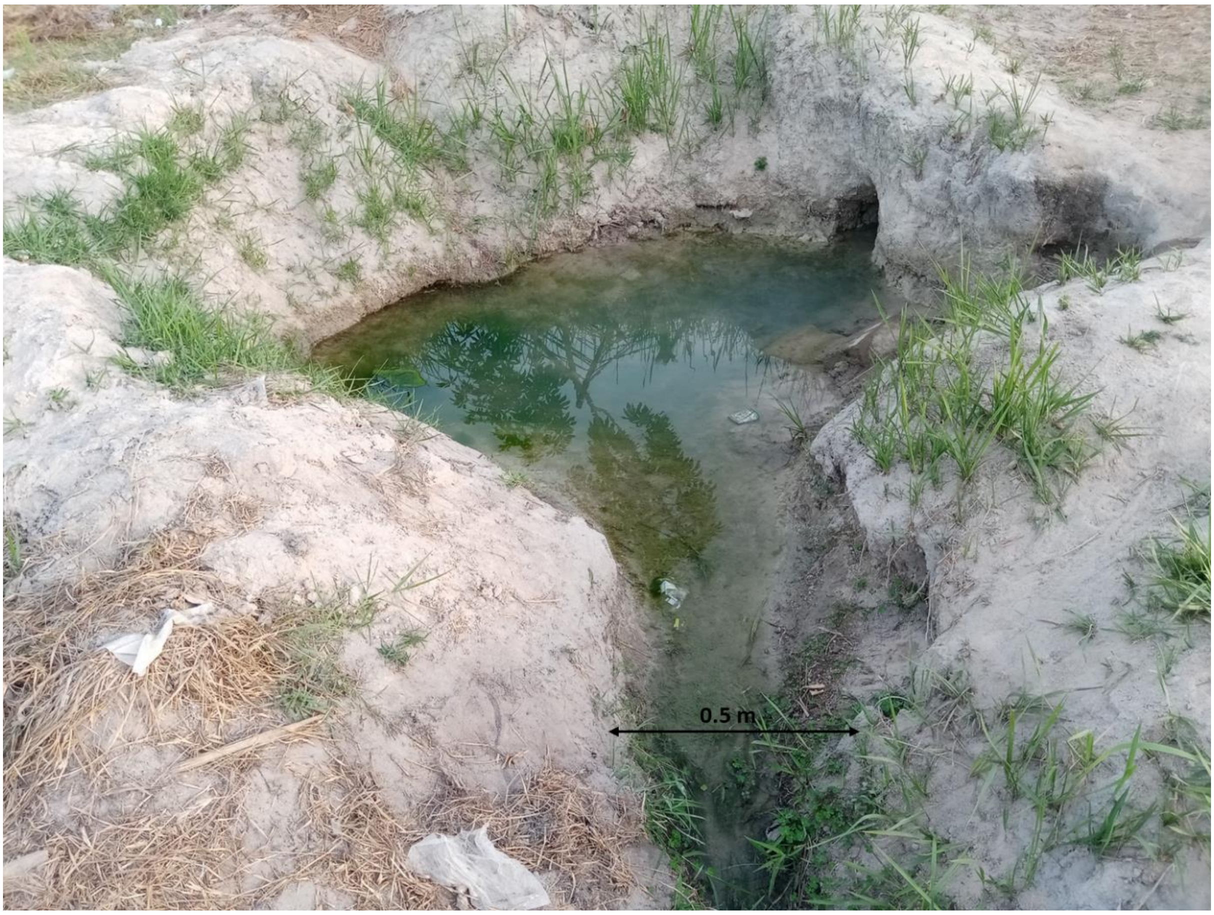

Appendix 8: Photograph showing a spring in the isalterites (location on Appendix 1 (Ap_8)).

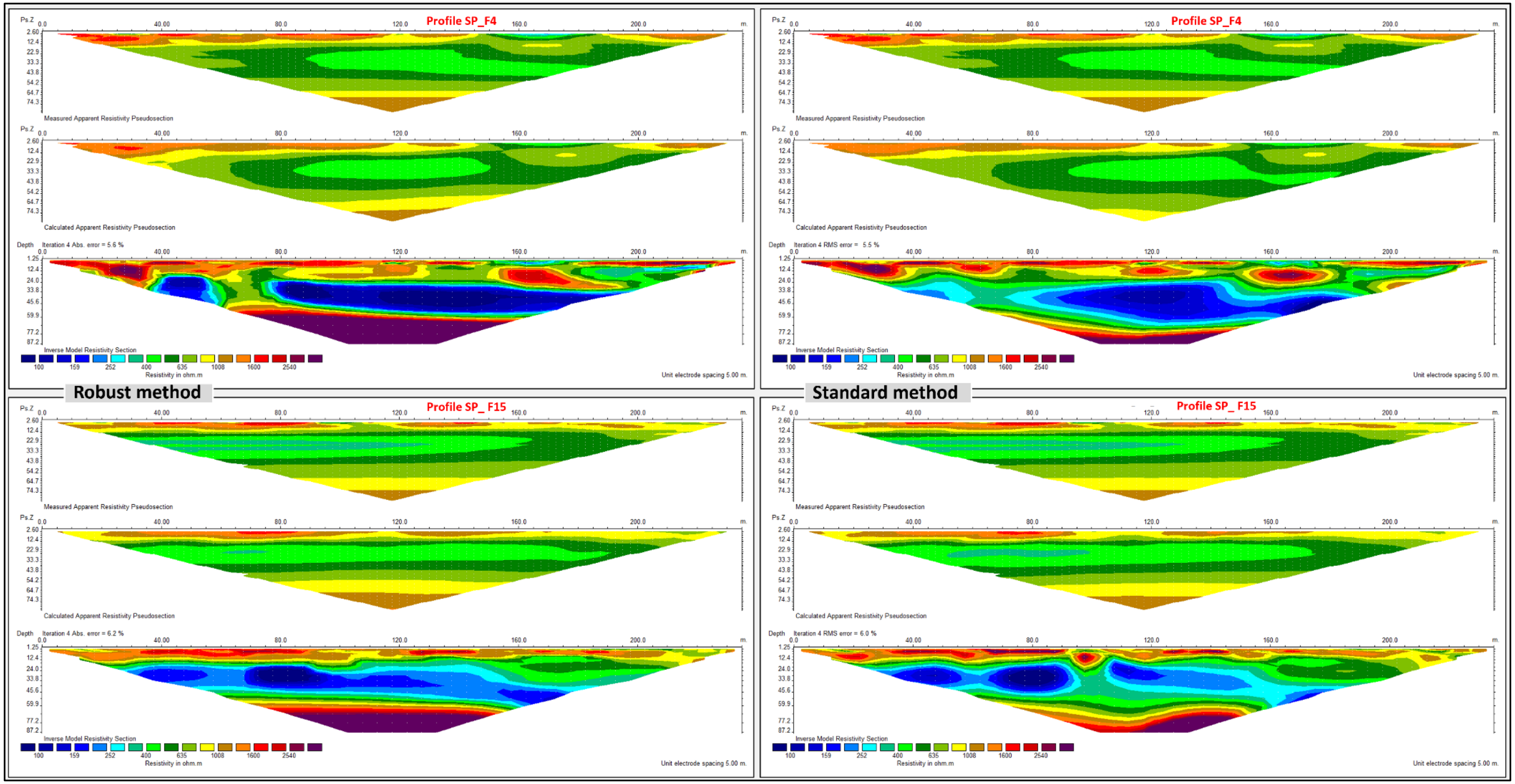

Appendix 9: Showing the pseudo-sections for the measured apparent resistivity, the calculated apparent resistivity and the inverted resistivity model for the SP_F4 and SP_F15 profiles made at the plateaus. On the left we have the ERT sections inverted using the robust method, and on the right the ERT sections inverted using the standard method.

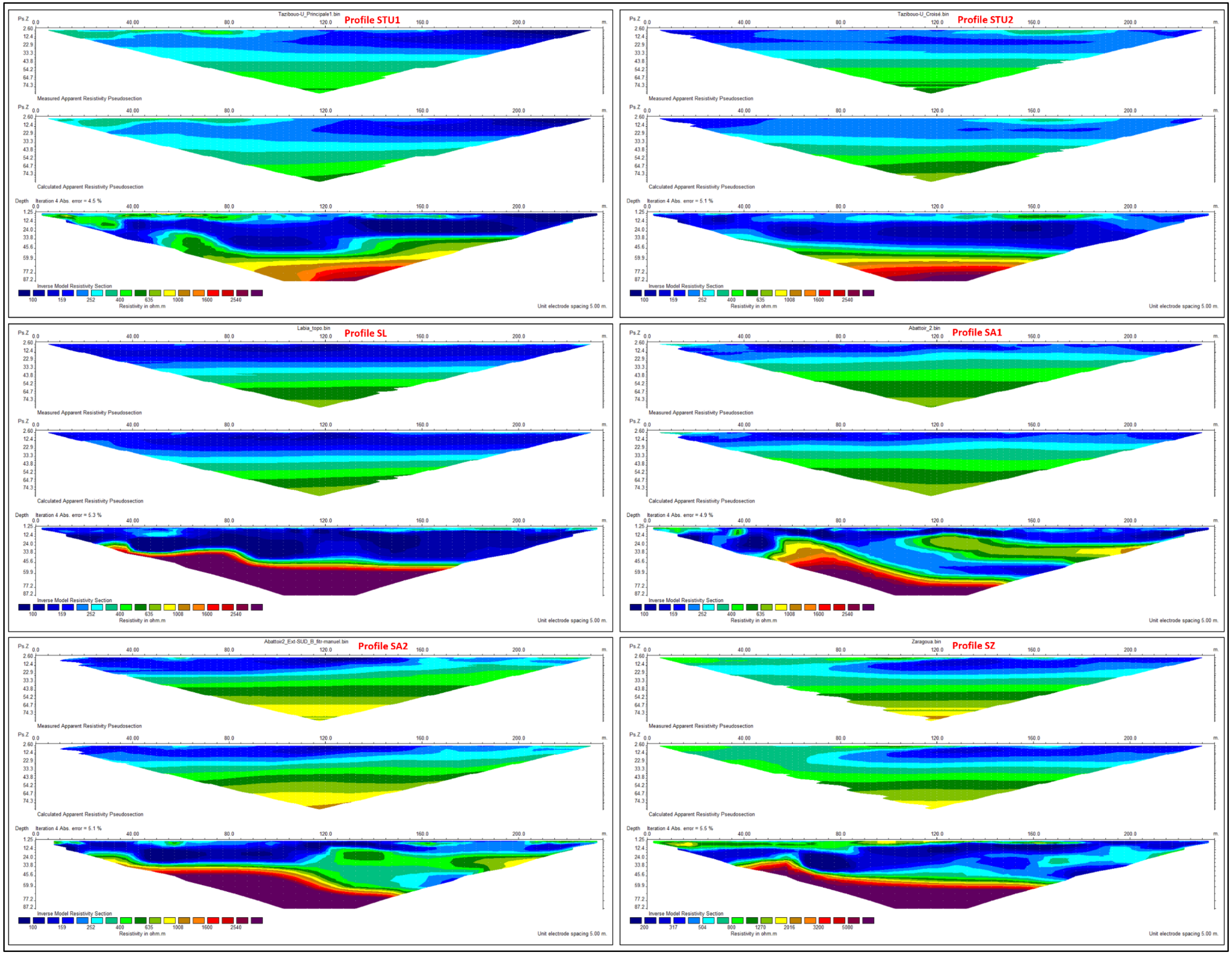

Appendix 10: Showing pseudo-sections for measured apparent resistivity, calculated apparent resistivity and inverted resistivity model for profiles made at springs and inverted with the robust method.
